# DNP-Enhanced Magic Angle Spinning Solid-State NMR Spectroscopy to Determine RNA–Ligand Interactions

**DOI:** 10.1021/jacs.5c17834

**Published:** 2025-12-30

**Authors:** Alexey Sudakov, Johanna Becker-Baldus, Konstantin S. Mineev, Anna Wacker, Hendrik R. A. Jonker, Felix Nussbaumer, Raphael Plangger, Clemens Glaubitz, Harald Schwalbe

**Affiliations:** † Institute for Organic Chemistry and Chemical Biology, Center for Biomolecular Magnetic Resonance (BMRZ), 9173 Goethe University Frankfurt am Main, Max-von-Laue-Str. 7, Frankfurt 60438, Germany; ‡ Institute for Biophysical Chemistry, Center for Biomolecular Magnetic Resonance (BMRZ), Goethe University Frankfurt am Main, Max-von-Laue-Str. 9, Frankfurt 60438, Germany; § Innotope, Center for Molecular Biosciences Innsbruck, Innrain 80/82, Innsbruck 6020, Austria

## Abstract

Understanding the molecular recognition underlying RNA–ligand complex formation is of key importance to explain the RNA regulatory function of riboswitches and to support the development of low-molecular-weight RNA binders as starting points for the development of RNA-targeting drugs. Here, we report magic angle spinning solid-state NMR spectroscopic studies enhanced by dynamic nuclear polarization (MAS-DNP) to determine the molecular recognition of a ligand–RNA riboswitch complex. We benchmarked different labeling strategies for four large RNAs (70–86 nt) of the aptamer domain of a 2′deoxyguanosine-sensing riboswitch from *Mesoplasma florum*. RNA samples were prepared either by chemoenzymatic approaches or by solid-phase chemical synthesis employing different labeling schemes of riboswitches of up to 86 nucleotides. RNA–ligand complexes were prepared by the addition of their cognate metabolite. We showed that nucleotide- and ligand-selective labeling are prerequisites for the MAS-DNP studies to reduce the NMR signal overlap present in such large RNAs. We further extended site-specific labeling to atom-specific labeling, which allowed us to derive the structure of the ligand-binding pocket, extending the application of 2D ^13^C,^15^N-TEDOR experiments. The work described here opens an avenue for the investigation of large RNA–ligand complexes by MAS-DNP.

## Graphical abstract

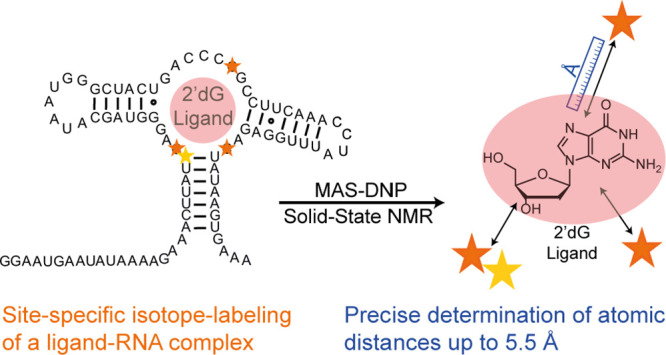

## Introduction

Detailed knowledge of RNA structure and dynamics at atomic resolution is essential to developing a molecular understanding of RNA function. Among noncoding RNAs, riboswitches are an intriguing example of mRNAs whose regulatory function is triggered by the interaction of a low-molecular-weight metabolite with untranslated regions of mRNAs. Riboswitches contain an aptamer domain that binds with high affinity and specificity to a metabolite. This binding induces allosteric conformational changes that can regulate transcription and translation.^1,2^

Structures of riboswitch–metabolite complexes were determined almost exclusively by X-ray crystallography.^3,4^ Alternative structural information is sparse despite recent impressive improvements in cryo-EM of riboswitches^5–7^ with resolutions down to 2.9 Å^7^ and notably also XFEL studies.^8–10^ In principle, NMR spectroscopy offers a powerful approach to probe RNA structure and conformational heterogeneity under near-physiological conditions; however, its application to determine solution structures of riboswitch–metabolite complexes has remained limited because these systems are often too large for solution NMR. These limitations have restricted NMR structural investigations of the RNA–ligand complexes to mapping the binding-induced chemical shift changes to identify the interaction sites; however, structural information at atomic resolution is missing. In order to overcome the size limits of solution NMR, divide-and-conquer approaches can be pursued, and successful applications showed, for example, that isolated RNA elements of the RNA genome of SARS-CoV-2 form autonomous folding units and retain their folding within larger RNA constructs of the entire 5′- and 3′-UTRs, respectively.^11^ Further, isotope-labeled riboswitches could be reconstituted to bind to the ribosome, revealing that only parts of the RNA chain are engaged within the ribosome, while the remainder stays flexible and can be observed by solution NMR in the riboswitch–ribosome complex.^12^

Next to solution NMR spectroscopy, it is increasingly recognized that solid-state NMR spectroscopy can augment the repertoire of structural methods. Initial solid-state NMR studies of RNAs by the Drobny group characterized ^31^P chemical shift anisotropies in tobacco mosaic virus^13^ and derived information about RNA dynamics by ^2^H solid-state NMR.^14^ The groups of Görlach and Ramachandran introduced solid-state NMR of uniformly ^13^C,^15^N-labeled RNAs,^15,16^ including trinucleotide repeat sequences.^17^ These early studies paved the way for the investigation of RNA–peptide complexes, in particular TAT-TAR by the groups of Drobny and Varani,^18,19^ and Rev-RRE in the Tycko group.^20^ Cherepanov et al.^21^ were able to transfer solution chemical shift assignments of the UUCG tetraloop, conducting ^13^C,^13^C radio-frequency-driven dipolar recoupling (RFDR)^22,23^ experiments. The Carlomagno group extended solid-state NMR to the study of RNA–small protein complexes, being the first to report a solid-state NMR structure of an RNA (PDB code: 2NOR) and of an RNA–protein complex (PDB code: 6TPH).^24–26^ Solid-state NMR for relatively small synthetic ribozymes and RNA aptamer–ligand or ribonucleoprotein complexes, enhanced by dynamic nuclear polarization techniques, were first reported by the groups of Corzilius, Suess, and Marchanka,^27–29^ while the Wang group conducted studies on the adenine–riboswitch complex.^30^ The determination of exact binding sites in RNA–ligand complexes at sub-Ångstrom precision and accuracy has, however, not yet been reported.

Here, we conduct DNP-enhanced magic angle spinning solid-state NMR studies (MAS-DNP) to determine exact distances between the metabolite 2′deoxyguanosine (2′dG) and the aptamer domain of the 2′dG-sensing riboswitch (2′dGsw) from *Mesoplasma florum* (*M. florum*). In both solution and solid-state NMR, determination of distance restraints in the region of 4–8 Å by NMR requires labeling of the biomolecular molecule of interest with ^13^C and ^15^N. While for proteins, uniform labeling schemes have allowed at least partial chemical-shift assignments for large folded (malate synthase G, 723 residues)^31^ and intrinsically disordered proteins (tau, 441 residues),^32,33^ the chemical shift dispersion of RNAs is low compared to proteins, which has put a stricter size limit to the study of RNAs by solution NMR. Of the ∼500 RNA NMR structures from the Nucleic Acid Database (NDB), only approximately 20 contain 60 or more nucleotides (nt), and the average size is 27 nt.^34^ Chemical shift assignments are mostly reported for H,N imino sites in hydrogen-bonded secondary structure elements of RNA.

With all aforesaid, it is apparent that site-specific incorporation of isotopically labeled nucleotides in the interesting sites of RNA’s structure is required to reduce signal overlap in NMR spectra. Several methodologies enable site-specific RNA labeling, including solid-phase chemical synthesis of either the entire RNA or short, labeled fragments. Following the latter approach, short fragments can be enzymatically ligated. Fragments can also be prepared by in vitro transcription and extended by 3′,5′-bisphospate nucleosides catalyzed by T4 RNA ligase using chemoenzymatic strategies that combine synthetic and enzymatic reaction steps.^35,36^

The incorporation of a single-labeled nucleotide dramatically reduced signal overlap in the 2D Transferred Echo DOuble Resonance (TEDOR) experiment and allowed us to obtain precise distances between the labeled nucleotide of 2′dGsw and its cognate ligand under DNP conditions.^37–39^ However, when using samples where all atoms were ^13^C,^15^N-labeled in the ligand and even only a single nucleotide of the riboswitch RNA, cross peaks in the TEDOR spectra were dominated by intramolecular cross peaks, and intermolecular distances between 2′dG and the 2′dGsw could not be determined. To determine the distances with an accuracy of the tenth of an Å, we thus prepared an atom-specifically labeled RNA–ligand complex. We benchmarked the TEDOR distances to structural information from crystallography and solution NMR, documenting a remarkably high-level consistency of various structural data.

## Results

### RNA Chemoenzymatic Synthesis, Splinted Ligation, and Solid-Phase Chemical Synthesis Provide the Site-Specific Cytidine or Adenosine Labeling of 2′dGsw^86^ and 2′dGsw^70^

Four samples of 2′dGsw were prepared, each containing a single site-specifically incorporated isotopically labeled nucleotide (Figure 1, Supplementary Chapter 1). Based on analysis of the crystal structure (PDB code: 3SKI) and the in-house solution structure, we selected cytidine nucleotides (C) at positions 26, 53, and 75 within an 86mer 2′dGsw^86^ construct and synthesized these site-specific labeled samples by chemoenzymatic and splinted ligation technologies. We further selected adenosine (A) 25 and prepared a sample by solid-phase chemical synthesis. All four nucleotides are involved in interactions with the ligand 2′dG: C75 forms a Watson–Crick hydrogen bond, C26 forms a hydrogen bond from its 2′–OH with its N7, C53 interacts with its sugar edge, and A25 is involved in stacking interactions.

**Figure 1:**
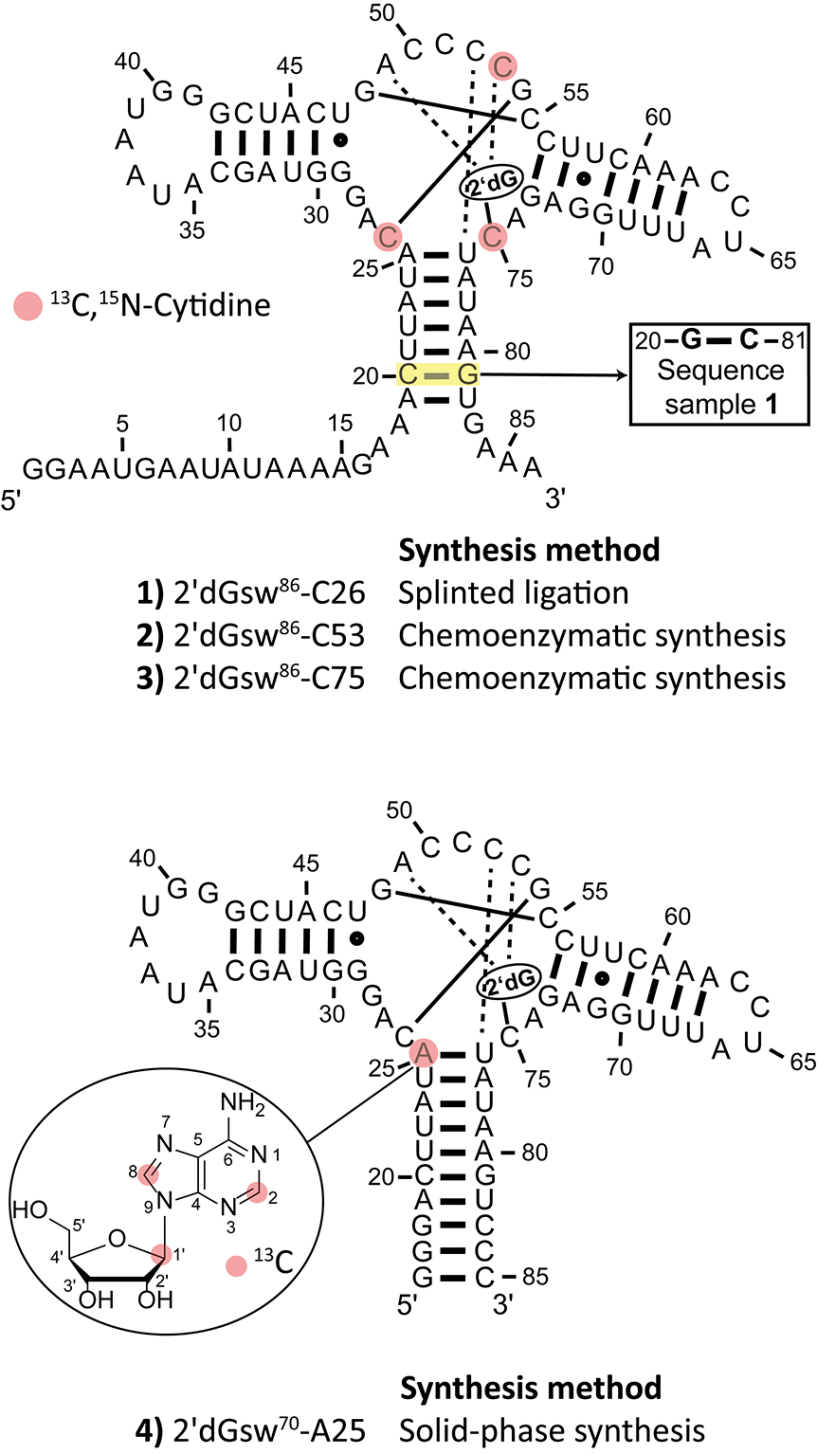
Secondary structure of the aptamer region of the 2′dGsw RNA. Samples 1–3 of the aptamer domain of the riboswitch 2′dGsw were prepared with a single site-specifically incorporated isotopically labeled ^13^C,^15^N-cytidine, highlighted in red. The synthesis methods for the preparation of the four individual samples are shown. The ligand 2′dG had the following labeling scheme: samples 1–3 (^13^C,^15^N) and sample 4 (^15^N).

For the chemoenzymatic preparations, we used two different strategies: RNA samples 2′dGsw^86^-C75 and 2′dGsw^86^-C53 were prepared (Supplementary Chapters 3.1–3.2), synthesizing ^13^C,^15^N-cytidine 3′,5′-bisphosphate (Supplementary Chapter 2) as the starting compound for T4 RNA ligase-catalyzed RNA 3′ extension, oxidation, dephosphorylation, and splinted ligation. For the preparation of the 2′dGsw^86^-C26 RNA (Supplementary Chapter 3.3), the cytidine in position 20 and the guanosine in position 81 were swapped compared to the native sequence. This swapping allowed preparation of the 2′dGsw^86^-C26 RNA with a splinted ligation as the only enzymatic step. Through the change of cytidine and guanosine, the first 27mer 2′dGsw^27^ RNA fragment for the splinted ligation had only a single cytidine in the sequence at position 26. When ^13^C,^15^N-cytidine triphosphate was added to the in vitro transcription of this fragment, only the desired cytidine at position 26 was isotopically labeled, increasing the yield significantly compared to the chemoenzymatic synthesis applied to prepare samples 2 and 3.

Furthermore, we chemically synthesized a 70mer 2′dGsw^70^ with a (1′,2,8)-^13^C-labeled adenosine at position 25 (A25) that is involved in P1 stabilization by binding to 2′dG (Supplmentary Chapter 3.4). Because yields in solid-phase synthesis scale down with the number of nucleotides due to linear synthesis, we removed the 5′- and 3′-terminal non-base-paired nucleotides. The purity of all samples was characterized by polyacrylamide gel electrophoresis (Figures S8, S10, S16, and S18). Despite the challenges associated with the solid-phase chemical synthesis of long RNAs, a pure product was obtained.

### Atom-Selective Isotope Labeling Allows Detection of Intermolecular Contacts by MAS-DNP TEDOR Experiments

First, a 2D ^13^C,^15^N-TEDOR MAS-DNP spectrum was recorded for the ^13^C,^15^N-labeled 2′dG in the absence of 2′dGsw^86^ (black signals) and with (red signals) 1.25-fold excess of riboswitch (Figure 2). The spectrum of the free ligand exhibited line broadening that we interpret to arise from inhomogeneous broadening at 100 K, most prominently observed for the C1′–N9 correlation peak. Strong chemical shift changes upon complex formation were observed for C8/N9, C8/N7, and C5/N7 cross peaks. The inhomogeneous broadening is reduced in the complex, which is in line with the formation of a conformationally homogeneous, specific RNA–ligand complex.

**Figure 2:**
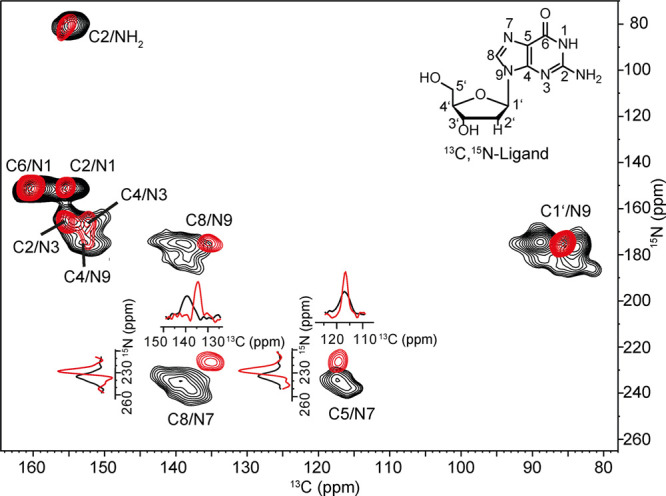
2D ^13^C,^15^N-TEDOR MAS-DNP spectrum of the ^13^C,^15^N-labeled deoxyguanosine 2′dG without (black) and with (red) 1.25 excess of unlabeled 2′dGsw^86^. 1D traces are shown for the C8/N7 and C5/N7 signals. The spectrum was recorded under MAS-DNP conditions at 100 K and 400 MHz (9.39 T) with a rotation speed of 10,417 Hz and a mixing time of 1.1 ms.

We then prepared three samples containing the 2′dGsw^86^ riboswitch, where either C26, C53, or C75 nucleotides and the ligand were uniformly ^13^C,^15^N isotope-labeled. For all samples, a 2-fold excess of ^13^C,^15^N isotope-labeled ligand was added to ensure the formation of the 2′dG-2′dGsw^86^ complex. The NMR spectra of the uniformly ^13^C,^15^N isotope-labeled samples are shown in Figure 3A–C. Correlation peaks induced by dipolar interactions of the RNA are annotated in red, and those of the ligand are annotated in black. Despite the different sequence positions (C26, C53, and C75), the chemical shifts of these three cytidines were mostly indistinguishable (Figure S27). This lack of chemical shift dispersion demonstrates that for an RNA sample in which all nucleotides are isotope-labeled (e.g., all cytidines), it does not seem likely that unambiguous distance data can be obtained in ^13^C–^15^N correlation spectra. The information content of ^13^C and ^15^N chemical shifts between different RNA target cytidines remained limited. The 2-fold excess of 2′dG over RNA gave rise to two sets of ligand signals representing the free and bound states of the ligand. The difference in chemical shifts between these two states showed that the conformations around the glycosidic torsion angle χ were likely different for free and bound 2′dG. Interestingly, we also observed two signals for the C1′/N1 correlation of C26 in the 2D ^13^C,^15^N-TEDOR spectra (Figure 3A). Detection of these two signals likely reflects a certain degree of heterogeneity for the ligand-binding pocket in the holo state of 2′dGsw^86^. In contrast, the C53 and C75 positions did not show such heterogeneity. For all three samples, where both the ligand and the RNA were uniformly labeled, we could only observe intramolecular correlations in the 2D ^13^C,^15^N-TEDOR spectra. Intermolecular correlation peaks, essential for distance determination within the complex, could not be observed.

**Figure 3:**
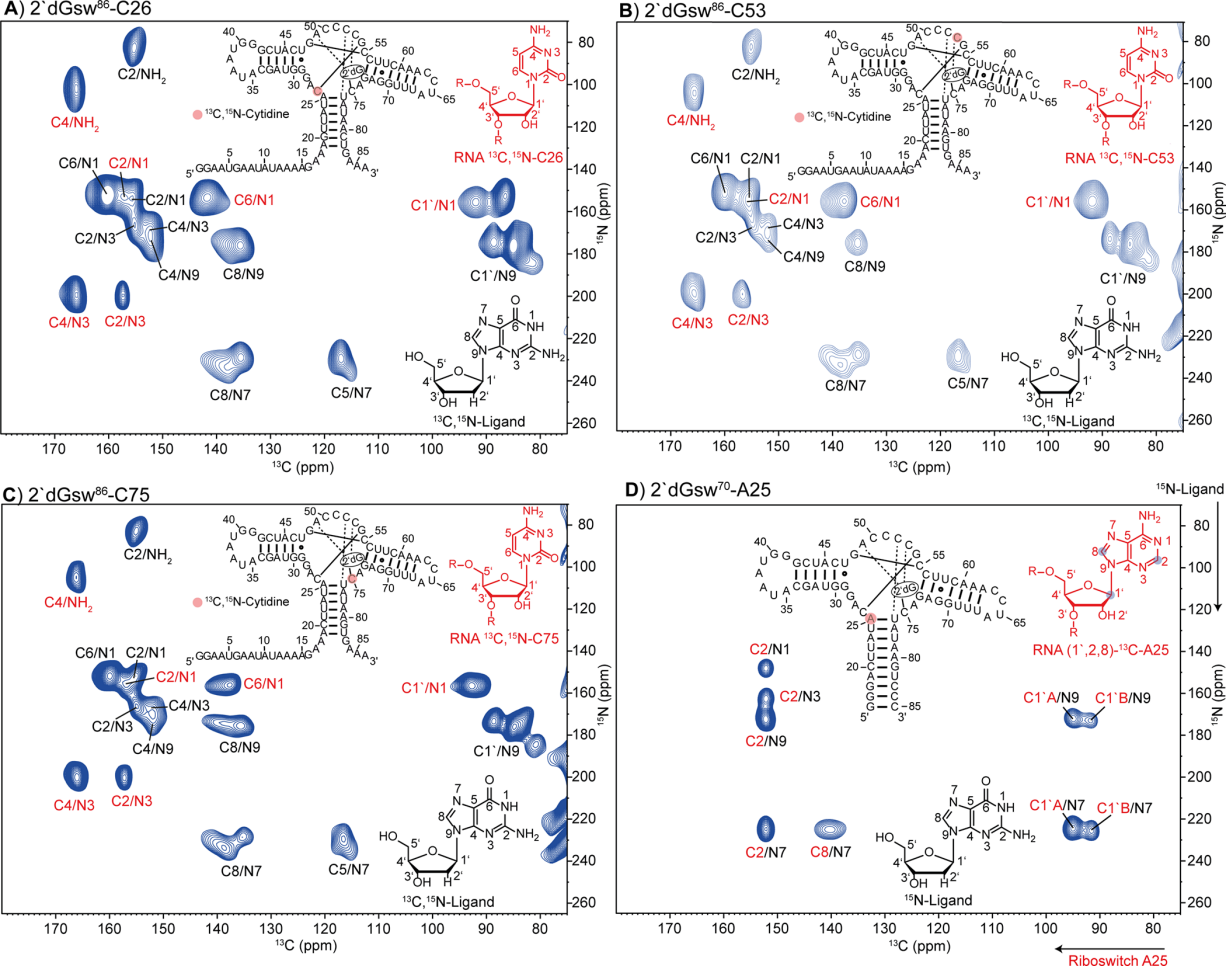
2D ^13^C,^15^N-TEDOR spectra of the complex between aptamer domain of the riboswitch (2′dGsw) and 2′deoxyguanosine (2′dG). (A–C) 2′dGsw^86^ labeled with ^13^C,^15^N at sequence positions C26/C53/C75, respectively, with 2 equiv of ^13^C,^15^N-labeled 2′dG. Signals annotated in red arise from 2′dGsw and signals annotated in black come from 2′dG. Signals annotated in red/black in spectrum D represent intermolecular ^13^C,^15^N correlations between 2′dG and 2′dGsw. (D) 2′dGsw^70 13^C-labeled at atoms C1′, C2, and C8 in adenosine 25 with 2 equiv of ^15^N-labeled 2′dG. The spectra were recorded under MAS-DNP conditions at 100 K and 400 MHz (9.39 T) with a rotation speed of 10,417 Hz and mixing times of 1.1 ms (A–C) and 20.4 ms (D).

We thus tested whether 2′dGsw samples could be obtained with sufficient purity and in sufficient chemical yields by solid-phase chemical synthesis. In combination with atom-selective labeling within a single nucleotide of the target RNA (^13^C) and the ligand (^15^N), we tailored the sample preparation to obtain the intermolecular TEDOR distances. We first prepared a sample containing the Watson–Crick acceptor nucleotide C75 in C1′, C6, D5 isotope-labeled form (Supplementary Chapter 3.5). In fact, we could detect weak TEDOR signals between the ligand and this nucleotide (Figure S25). Then, we labeled A25 with ^13^C-labeled at the carbon atoms C1′, C2, and C8. Using the exclusively ^15^N-labeled ligand, signals arising from intermolecular ^13^C,^15^N dipolar interactions could be detected. In Figure 3D, the 2D ^13^C,^15^N-TEDOR spectrum of the 2′dGsw^70^-A25 RNA with 2 equiv of the ^15^N-ligand is shown. All correlation peaks in the spectrum of ^13^C-labeled sites C1′, C2, and C8 of the adenosine-labeled riboswitch to the ^15^N-labeled sites N9, N7, N3, and N1 of the ligand were used for distance determination between A25 and the ligand as described in the following section.

### MAS-DNP Distances Provide a Single Possible Orientation of 2′dG in Complex with the Riboswitch

All samples for MAS-DNP experiments were prepared by adding 50% (v) d8-glycerol and the polarizing agent AsymPolPOK at a final concentration of 10 mM.^40,41^ The MAS-DNP measurements were carried out, as described in Supplementary Chapter 4, and DNP enhancements in the range of 64–100 were obtained for the different samples. To determine distances between the atoms C1′, C2, and C8 of A25 in the 2′dGsw^70^ RNA and the nitrogen atoms within the nucleobase of 2′dG, we recorded 2D ^13^C,^15^N-TEDOR spectra with variable mixing times (Table S21). We reached the maximum of the build-up curves for all signals shown in the spectrum of the 2′dGsw^70^ RNA (Figure 3D). Recording these build-up curves enabled precise distance calculations. The fit of the build-up curves and the simultaneous measurement of multiple carbon–nitrogen distances (Figure 4A) were performed, as described by Jaroniec et al.^38^ (eq 10), using Wolfram Mathematica as the simulation software package (Figure S23). All signals shown in Figure 3D were used in the fit of the build-up curves. The intensities of the other cross peaks, including all signals involving N2, were very weak at all recorded mixing times and result from very long distances, which could not be quantified. Comparison of the TEDOR-derived distances with data from the crystal structure (PDB code: 3SKI) and a solution-NMR-derived structural model of the binding pocket (Supplementary Chapter 5) determined by us^42^ revealed that while the distances are very similar, three (C2/N7, C8/N7, C1′/N7) out of seven TEDOR-derived distances are longer than the distances derived from X-ray (Figure 4B). For all three of these distances, this increase is in agreement with the distances found in the solution NMR model. We then calculated possible orientations of 2′dG and A25 (Figure 4C), which allowed a precise positioning of the ligand with respect to A25 in the single possible orientation (RMSD of the ligand coordinates equals 0.8 Å). The obtained RMSD is better than the X-ray resolution (2.3 Å) and is less than the uncertainty of the NMR structure (RMSD of the ligand coordinates, when A25 nucleobases are aligned, equals 1.4 Å). The precision of the ligand positioning could be further improved using integrative structural biology by combining TEDOR distances with the information from the other techniques (solution NMR or X-ray). In this manner, taking into account the X-ray structure of 2′dGsw^70^ provides the single possible orientation of guanine with an RMSD of 0.1 Å.

**Figure 4:**
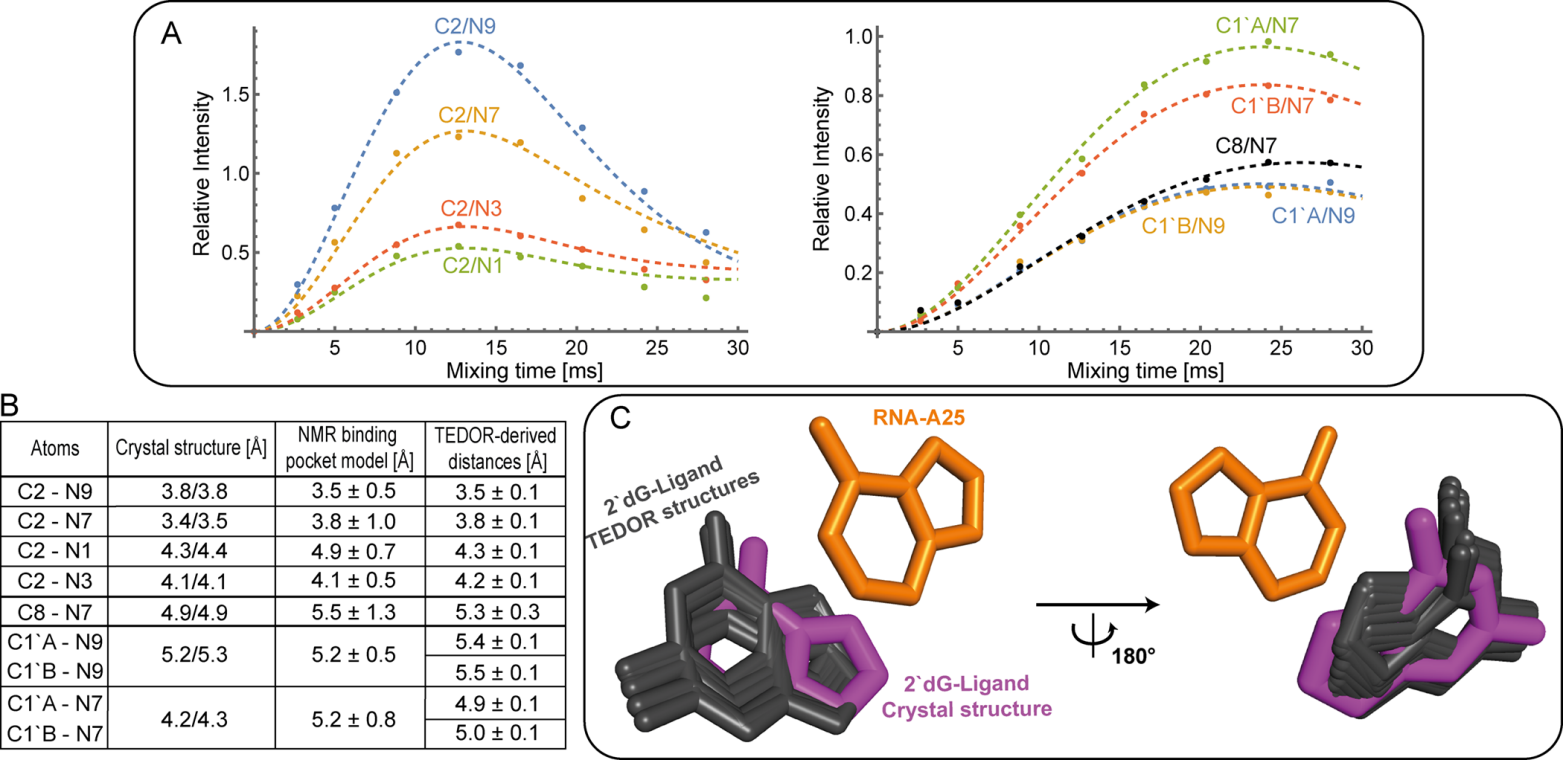
(A) TEDOR cross-peak intensities were measured for C1′, C2, and C8 of the adenosine (^13^C) and the N1, N3, N7, and N9 of the ligand (^15^N). Approximation of the experimental data by theoretical dependence is shown by dashed lines. The range of acceptable fits in a 95% confidence interval is shown in Figure S24. (B) Comparison of the distances determined here with the parameters of X-ray spectroscopy (3SKI) and the solution NMR structures. The crystal structure contains two RNA ligand complexes; therefore, two distances are shown in the table. For the solution NMR-derived binding pocket model, averaged distances and their respective errors are given. For the TEDOR-derived distances, the reported errors corresponding to the 95% confidence interval stem from fitting the experimental data to the theoretical cross-peak intensities. (C) Comparison of the crystal structure (magenta) and possible conformations based on the TEDOR-derived distances (gray) between A25 and the ligand.

## Discussion

### Comparison of Methods for Site-Specific Incorporation of Isotopically Labeled Nucleotides into RNA

We prepared four different samples using three different approaches with sample amounts ranging between 12 and 110 nmol. These approaches are solid-phase chemical synthesis, chemoenzymatic synthesis, and splinted ligation. All these techniques are compatible with both the site-specific and atom-selective labeling; however, they are different in terms of yields, costs, and time consumption. At the start of the work, it was not obvious whether RNA samples of sufficient amount and purity could be obtained by solid-phase chemical RNA synthesis for an RNA with 70 nucleotides. Thus, we can report here that high quantities of high purity can be obtained by custom synthesis conducted by one of us (Innotope). The solid-phase synthesis provided us with high yields for an RNA with 70 nucleotides. However, synthesis of longer RNAs remains problematic, as solid-phase synthesis is a linear synthesis, whose yields scale down with RNA length. In contrast, the chemoenzymatic approaches implemented in the current work are not limited by the length of the final RNA construct and are therefore more versatile while more time-consuming. One could estimate the costs of chemoenzymatic ligation as 97 USD per nmol, provided that the T4 RNA ligase 2 is expressed in-house. The cost of the single-step splinted ligation could be estimated as 8 USD/nmol. These amounts are mostly independent of the length of the construct. The costs of the solid-phase chemical synthesis, in turn, would grow with the length and reach 24 USD per nmol for 70 nucleotides. According to the experience of Innotope, this length lies on the border of the applicability of commercial solid-phase synthesis. Longer RNA constructs could still be synthesized, but neither purity nor overall success can be guaranteed. Considering the time expenses of the ligation techniques, we state that solid-phase chemical synthesis is the technique of choice for the shorter site-specifically labeled RNAs, with a current upper limit at around 70–80 nucleotides. For longer constructs, we recommend using the solid-phase synthesis of atom-specifically labeled oligonucleotides followed by the single-step splinted ligation. Chemoenzymatic ligation would be a method of choice for longer RNAs that exceed 150–200 nucleotides. Our work further documents that nucleotides can be incorporated at any position within the target RNA using all of the tested approaches. Regardless of the differences in the sample amount, the signal-to-noise enhancements offered by DNP are sufficient for the precise determination of atomic distances up to 5.5 Å.

### Advantages and Challenges of MAS-DNP

Here, we provide a proof-of-concept that MAS-DNP can be used to investigate the structure and dynamics of RNA–ligand complexes. MAS-DNP can measure distances with a precision of 0.1–0.2 Å between heteronuclei directly. We note that developments for the magic angle solid-state NMR measurement of ^1^H–^1^H distances are currently pursued. These studies have, however, not been extended to MAS-DNP.^43^ This precision is better than the distances between heavy atoms in X-ray crystallography data. While solution NMR can yield similar precise distances for selected ^1^H–^1^H pairs, care has to be taken to account for spin diffusion.^44–46^

MAS-DNP measurements provide force-field-independent distances between the heteroatoms of nucleobases engaged in the base pairing interactions, valuable information that cannot be obtained in solution. For specific RNA–ligand complexes, problems of broad signals and complicated assignments, inherent to MAS-DNP, can be overcome by advanced isotope labeling schemes.

Uniform labeling can yield high signal intensities with DNP across the spectrum but will suffer from limited ^13^C and ^15^N chemical shift dispersion in the RNA’s nucleobase NMR signals. Site-specific labeling simplifies the assignment dramatically, and the atom-selective labeling reduces the number of dipolar-coupled spins within the nucleobase of the site-specifically labeled target RNA. As documented here, using ^13^C labeling for a single nucleotide in the RNA riboswitch and ^15^N labeling for the ligand tailors the atom-selective labeling patterns to the requirements of the TEDOR experiment. The removal of short ^13^C–^15^N distances is mandatory for the measurement of long distances due to the dipolar truncation effect. Application of TEDOR to the atom-specific labeled RNA sample provided us with seven distances, determined with a precision of 0.1 Å (one distance with a precision of 0.3 A). The TEDOR-derived distances deviate from the X-ray structure by no more than 0.5 Å, which can be considered to be in good agreement, taking into account the experimental errors and resolution of the X-ray structure (2.3 Å). This set of restraints allowed determining the precise orientation of the nucleobase of deoxyguanosine with respect to the A25 nucleobase (RMSD = 0.8Å). Furthermore, considering the X-ray-derived RNA conformation, one could further reduce the ambiguity and arrive at a single well-defined conformation of the ligand. Theoretically, it is possible to measure even longer distances with longer mixing times. However, signal intensity loss, caused by relaxation, must be taken into account with longer mixing times.

## Conclusions

Up to now, MAS-DNP solid-state NMR has not progressed to RNA–ligand complex structure determination. Here, we show that for riboswitch RNA that forms a highly specific complex with a ligand with low dissociation constants, solid-state spectra can be obtained with highly reduced inhomogeneous line broadening. Site- and atom-specific labeling schemes and alternative labeling of the riboswitch with ^13^C and of the ligand with ^15^N allowed us to measure precise and accurate intermolecular distances between multiple ^13^C and ^15^N spin pairs.

Taking steric clashes in the NMR and crystal structures into account, the distances in a single sample are sufficient to position the ligand within the riboswitch binding pocket in an unambiguous manner. Our approach expands the variety of methods for determining RNA–ligand complexes, and the insights described here on optimal sample design should make such studies part of the structural biology portfolio. Information about RNA–ligand complexes is important to understand RNA-based regulatory roles of ligands in targeting RNAs and also for the structure-based discovery of RNA-targeting drugs. With the preparation methodology described here, neither RNA size nor labeling remains a limiting factor.

## Supplementary Material



## Data Availability

Experimental raw data are available at 10.25716/gude.126s-qxrm (Goethe University Data Repository, GUDe).

## Notes

Supplementary synthesis and experiments mentioned in the main text (Supporting Information I) (PDF)

## Abbreviations

2′dG: 2′deoxyguanosine
2′dGsw: 2′dG-sensing riboswitch
Å: Angstrom
cryo-EM: cryogenic electron microscopy
DNP: dynamic nuclear polarization
Hz: hertz
K: kelvin
MAS: magic angle spinning
ms: millisecond
NMR: nuclear magnetic resonance
nt: nucleotides
PDB: Protein Data Bank
RMSD: root-mean-square deviation
SARS-CoV-2: Severe Acute Respiratory Syndrome Coronavirus-2
T: Tesla
USD: United States dollar
UTR: untranslated region
XFEL: X-ray free-electron laser

## References

[1] Kavita K., Breaker R. R. (2023). Discovering Riboswitches: The Past and the Future. Trends Biochem. Sci..

[2] Scull C. E., Dandpat S. S., Romero R. A., Walter N. G. (2021). Transcriptional
Riboswitches Integrate Timescales for Bacterial Gene Expression Control. Front Mol. Biosci.

[3] Serganov A., Nudler E. (2013). A Decade of Riboswitches. Cell.

[4] Zhang J., Jones C. P., Ferré-D’Amaré A. R. (2014). Global
Analysis of Riboswitches by Small-Angle X-Ray Scattering and Calorimetry. Biochim Biophys Acta Gene Regul Mech.

[5] Zhang K., Li S., Kappel K., Pintilie G., Su Z., Mou T. C., Schmid M. F., Das R., Chiu W. (2019). Cryo-EM Structure of
a 40 KDa SAM-IV Riboswitch RNA at 3.7 Å Resolution. Nat. Commun..

[6] Ding J., Deme J. C., Stagno J. R., Yu P., Lea S. M., Wang Y. X. (2023). Capturing Heterogeneous Conformers
of Cobalamin Riboswitch
by Cryo-EM. Nucleic Acids Res..

[7] Jaspersen, N. ; Prajapati, J. ; Singhal, A. ; Sanbonmatsu, K. Cryo-EM Reveals Remodeling of a Tandem Riboswitch at 2.9 Å Resolution. Res. Sq. 2025. 10.21203/rs.3.rs-6422592/v1.

[8] Stagno J. R., Liu Y., Bhandari Y. R., Conrad C. E., Panja S., Swain M., Fan L., Nelson G., Li C., Wendel D. R., White T. A., Coe J. D., Wiedorn M. O., Knoska J., Oberthuer D., Tuckey R. A., Yu P., Dyba M., Tarasov S. G., Weierstall U., Grant T. D., Schwieters C. D., Zhang J., Ferré-D’Amaré A. R., Fromme P., Draper D. E., Liang M., Hunter M. S., Boutet S., Tan K., Zuo X., Ji X., Barty A., Zatsepin N. A., Chapman H. N., Spence J. C. H., Woodson S. A., Wang Y. X. (2017). Structures of Riboswitch RNA Reaction
States by Mix-and-Inject XFEL Serial Crystallography. Nature.

[9] Stagno, J. R. ; Knoska, J. ; Chapman, H. N. ; Wang, Y.-X. Mix-and-Inject Serial Femtosecond Crystallography to Capture RNA Riboswitch Intermediates; Methods in Molecular Biology; Humana: New York, NY, 2023; Vol. 2568. 10.1007/978-1-0716-2687-0_16 36227573

[10] Lee H. K., Conrad C. E., Magidson V., Heinz W. F., Pauly G., Yu P., Ramakrishnan S., Stagno J. R., Wang Y. X. (2022). Developing Methods
to Study Conformational Changes in RNA Crystals Using a Photocaged
Ligand. Front Mol. Biosci.

[11] Wacker A., Weigand J. E., Akabayov S. R., Altincekic N., Bains J. K., Banijamali E., Binas O., Castillo-Martinez J., Cetiner E., Ceylan B., Chiu L. Y., Davila-Calderon J., Dhamotharan K., Duchardt-Ferner E., Ferner J., Frydman L., Fürtig B., Gallego J., Tassilo Grün J., Hacker C., Haddad C., Hähnke M., Hengesbach M., Hiller F., Hohmann K. F., Hymon D., de Jesus V., Jonker H., Keller H., Knezic B., Landgraf T., Löhr F., Luo L., Mertinkus K. R., Muhs C., Novakovic M., Oxenfarth A., Palomino-Schätzlein M., Petzold K., Peter S. A., Pyper D. J., Qureshi N. S., Riad M., Richter C., Saxena K., Schamber T., Scherf T., Schlagnitweit J., Schlundt A., Schnieders R., Schwalbe H., Simba-Lahuasi A., Sreeramulu S., Stirnal E., Sudakov A., Tants J. N., Tolbert B. S., Vögele J., Weiß L., Wirmer-Bartoschek J., Wirtz Martin M. A., Wöhnert J., Zetzsche H. (2020). Secondary Structure
Determination of Conserved SARS-CoV-2
RNA Elements by NMR Spectroscopy. Nucleic Acids
Res..

[12] de
Jesus V., Qureshi N. S., Warhaut S., Bains J. K., Dietz M. S., Heilemann M., Schwalbe H., Fürtig B. (2021). Switching
at the Ribosome: Riboswitches Need RProteins as Modulators to Regulate
Translation. Nat. Commun..

[13] Cross T. A., Opella S. J., Caspar D. L. D. (1983). 31p
Nuclear Magnetic Resonance of
the RNA in Tobacco Mosaic Virus. J. Mol. Biol..

[14] Wang A. C., Kennedy M. A., Reid B. R., Drobny G. P. (1994). A Solid-State 2H
NMR Investigation of Purine Motion in a 12 Base Pair RNA Duplex. J. Magn. Reson..

[15] Leppert J., Heise B., Ramachandran R. (2001). Orientational Information from TEDOR
Spectral Sidebands. Solid State Nucl. Magn.
Reson..

[16] Leppert J., Urbinati C. R., Häfner S., Ohlenschläger O., Swanson M. S., Görlach M., Ramachandran R. (2004). Identification
of NH···N Hydrogen Bonds by Magic Angle Spinning Solid
State NMR in a Double-Stranded RNA Associated with Myotonic Dystrophy. Nucleic Acids Res..

[17] Riedel K., Herbst C., Häfner S., Leppert J., Ohlenschläger O., Swanson M. S., Görlach M., Ramachandran R. (2006). Constraints
on the Structure of (CUG) 97 RNA from Magic-Angle-Spinning Solid-State
NMR Spectroscopy. Angew. Chem..

[18] Olsen G. L., Edwards T. E., Deka P., Varani G., Sigurdsson S. T., Drobny G. P. (2005). Monitoring Tat Peptide Binding to TAR RNA by Solid-State
31P-19F REDOR NMR. Nucleic Acids Res..

[19] Huang W., Varani G., Drobny G. P. (2010). 13C/15N-19F
Intermolecular REDOR
NMR Study of the Interaction of TAR RNA with Tat Peptides. J. Am. Chem. Soc..

[20] Havlin R. H., Blanco F. J., Tycko R. (2007). Constraints
on Protein Structure
in HIV-1 Rev and Rev-RNA Supramolecular Assemblies from Two-Dimensional
Solid State Nuclear Magnetic Resonance. Biochemistry.

[21] Cherepanov A. V., Glaubitz C., Schwalbe H. (2010). High-Resolution Studies of Uniformly13C,15N-Labeled
RNA by Solid-State NMR Spectroscopy. Angewandte
Chemie - International Edition.

[22] Bennett A. E., Rienstra C. M., Griffiths J. M., Zhen W., Lansbury P. T., Griffin R. G. (1998). Homonuclear Radio
Frequency-Driven Recoupling in Rotating
Solids. J. Chem. Phys..

[23] Griffiths J. M., Lakshmi K. V., Bennett A. E., Raap J., van der
Wielen C. M., Lugtenburg J., Herzfeld J., Griffin R. G. (1994). Dipolar
Correlation NMR Spectroscopy of a Membrane Protein. J. Am. Chem. Soc..

[24] Jehle S., Falb M., Kirkpatrick J. P., Oschkinat H., Van Rossum B. J., Althoff G., Carlomagno T. (2010). Intermolecular
Protein-RNA Interactions Revealed by 2D31P- 15N Magic Angle Spinning
Solid-State NMR Spectroscopy. J. Am. Chem. Soc..

[25] Asami S., Rakwalska-Bange M., Carlomagno T., Reif B. (2013). Protein-RNA Interfaces
Probed by 1H-Detected MAS Solid-State NMR Spectroscopy. Angewandte Chemie - International Edition.

[26] Marchanka A., Simon B., Carlomagno T. (2013). A Suite of
Solid-State NMR Experiments
for RNA Intranucleotide Resonance Assignment in a 21 KDa Protein-RNA
Complex. Angewandte Chemie - International Edition.

[27] Daube D., Vogel M., Suess B., Corzilius B. (2019). Dynamic Nuclear
Polarization on a Hybridized Hammerhead Ribozyme: An Explorative Study
of RNA Folding and Direct DNP with a Paramagnetic Metal Ion Cofactor. Solid State Nucl. Magn. Reson..

[28] Aladin V., Vogel M., Binder R., Burghardt I., Suess B., Corzilius B. (2019). Complex Formation
of the Tetracycline-Binding
Aptamer Investigated by Specific Cross-Relaxation under DNP. Angew. Chem..

[29] Aladin V., Sreemantula A. K., Biedenbänder T., Marchanka A., Corzilius B. (2023). Specific Signal
Enhancement on an RNA-Protein Interface
by Dynamic Nuclear Polarization. Chem.—Eur.
J..

[30] Zhao S., Li X., Wen Z., Zou M., Yu G., Liu X., Mao J., Zhang L., Xue Y., Fu R., Wang S. (2022). Dynamics of
Base Pairs with Low Stability in RNA by Solid-State Nuclear Magnetic
Resonance Exchange Spectroscopy. iScience.

[31] Tugarinov V., Kay L. E. (2003). Ile, Leu, and Val
Methyl Assignments of the 723-Residue
Malate Synthase G Using a New Labeling Strategy and Novel NMR Methods. J. Am. Chem. Soc..

[32] Mukrasch M. D., Bibow S., Korukottu J., Jeganathan S., Biernat J., Griesinger C., Mandelkow E., Zweckstetter M. (2009). Structural Polymorphism of 441-Residue
Tau at Single
Residue Resolution. PLoS Biol..

[33] Nováček J., Žídek L., Sklenář V. (2014). Toward Optimal-Resolution
NMR of Intrinsically Disordered Proteins. J.
Magn. Reson..

[34] Barton S., Heng X., Johnson B. A., Summers M. F. (2013). Database
Proton
NMR Chemical Shifts for RNA Signal Assignment and Validation. J. Biomol NMR.

[35] Keyhani S., Goldau T., Blümler A., Heckel A., Schwalbe H. (2018). Chemo-Enzymatic
Synthesis of Position-Specifically Modified RNA for Biophysical Studies
Including Light Control and NMR Spectroscopy. Angewandte Chemie - International Edition.

[36] Sudakov A., Knezic B., Hengesbach M., Fürtig B., Stirnal E., Schwalbe H. (2023). Site-Specific Labeling
of RNAs with
Modified and 19F-Labeled Nucleotides by Chemo-Enzymatic Synthesis. Chem.—Eur. J..

[37] Hing A. W., Vega S., Schaeffer J. (1993). Measurement of Heteronuclear Dipolar
Coupling by Transferred-Echo Double-Resonance NMR. J. Magn. Reson..

[38] Jaroniec C. P., Filip C., Griffin R. G. (2002). 3D TEDOR
NMR Experiments for the
Simultaneous Measurement of Multiple Carbon-Nitrogen Distances in
Uniformly 13C,15N-Labeled Solids. J. Am. Chem.
Soc..

[39] Wilson C. B., Tycko R. (2024). Optimization of 15N–13C Double-Resonance NMR Experiments under
Low Temperature Magic Angle Spinning Dynamic Nuclear Polarization
Conditions. J. Magn. Reson..

[40] Becker-Baldus J., Yeliseev A., Joseph T. T., Sigurdsson S. T., Zoubak L., Hines K., Iyer M. R., van den
Berg A., Stepnowski S., Zmuda J., Gawrisch K., Glaubitz C. (2023). Probing the
Conformational Space of the Cannabinoid Receptor 2 and a Systematic
Investigation of DNP-Enhanced MAS NMR Spectroscopy of Proteins in
Detergent Micelles. ACS Omega.

[41] Mentink-Vigier F., Marin-Montesinos I., Jagtap A. P., Halbritter T., Van Tol J., Hediger S., Lee D., Sigurdsson S. T., De Paëpe G. (2018). Computationally Assisted Design of Polarizing Agents
for Dynamic Nuclear Polarization Enhanced NMR: The AsymPol Family. J. Am. Chem. Soc..

[42] Wacker, A. B. Struktur, Dynamik und Funktion des 2’-Desoxyguanosin-Riboschalters. PhD-Thesis, Goethe University Frankfurt am Main: Frankfurt am Mai, 2012.

[43] Grohe K., Nimerovsky E., Singh H., Vasa S. K., Söldner B., Vögeli B., Rienstra C. M., Linser R. (2019). Exact Distance Measurements
for Structure and Dynamics in Solid Proteins by Fast-Magic-Angle-Spinning
NMR. Chem. Commun..

[44] Chiarparin E., Pelupessy P., Cutting B., Eykyn T. R., Bodenhausen G. (1999). Normalized
One-Dimensional NOE Measurements in Isotopically Labeled Macromolecules
Using Two-Way Cross-Polarization. J. Biomol
NMR.

[45] Bonvin A. M. J. J., Rullmann J. A. C., Lamerichs R. M. J. N., Boelens R., Kaptein R. (1993). “Ensemble” Iterative
Relaxation Matrix Approach: A New NMR Refinement Protocol Applied
to the Solution Structure of Crambin. Proteins:
Struct., Funct., Bioinf..

[46] Orts J., Vögeli B., Riek R. (2012). Relaxation Matrix Analysis of Spin
Diffusion for the NMR Structure Calculation with ENOEs. J. Chem. Theory Comput.

