# Supplementary material for: DNP-Enhanced Magic Angle Spinning Solid-State NMR Spectroscopy to Determine RNA–Ligand Interactions
Source: J Am Chem Soc. 2025 Dec 30;148(1):1329–36. doi: 10.1021/jacs.5c17834 (PMC12814176; doi:10.1021/jacs.5c17834)
Supplement: Supplementary file 1 [file ja5c17834_si_001.pdf]

DNP-Enhanced Magic Angle Spinning Solid-State NMR Spectroscopy to Determine RNA-Ligand Interactions

Alexey Sudakov[1], Johanna Becker-Baldus[2], Konstantin S. Mineev[1], Anna Wacker[1], Hendrik R. A. Jonker[1], Felix Nussbaumer[3], Raphael Plangger[3], Clemens Glaubitz[2] and Harald Schwalbe[1,*]

[1]Institute for Organic Chemistry and Chemical Biology, Center for Biomolecular Magnetic Resonance (BMRZ), Goethe University Frankfurt am Main, Frankfurt 60438, Max-von-Laue-Str. 7, Germany

[2]Institute for Biophysical Chemistry, Center for Biomolecular Magnetic Resonance (BMRZ), Goethe University Frankfurt am Main, Frankfurt 60438, Max-von-Laue-Str. 9, Germany

[3]Innotope, Center for Molecular Biosciences Innsbruck, Innrain 80/82, 6020 Innsbruck, Austria

*To whom correspondence should be addressed. Tel: +49 (0)69 / 798-29737; Fax: +49 (0)69 / 798-29515; Email: schwalbe@nmr.uni-frankfurt.de

KEYWORDS: MAS-DNP solid-state NMR, RNA, Distance determination, 2D-TEDOR, Site-specific labeling, Riboswitch

Table of Contents

Experimental Section 2

1. DNA and RNA sequences of the 2'dGsw^{86}-C26/C53/C75 and 2'dGsw^{70}-A25 2
2. Synthesis of the ^{13}C,^{15}N-cytidine 3',5'-bisphosphate 6
   - 2.1 General working techniques 6
   - 2.2 Synthesis of the ^{13}C,^{15}N-cytidine 3',5'-bisphosphate 6
   - 2.3 Characterization of synthesized ^{13}C,^{15}N-cytidine 3',5'-bisphosphate 7
3. Preparation of the site-specific labeled RNAs 2'dGsw^{86}-C26/C53/C75 and 2'dGsw^{25}-A25 10
   - 3.1 Preparation of the 2'dGsw^{86}-C75 RNA with the chemoenzymatic synthesis 10
   - 3.2 Preparation of the 2'dGsw^{86}-C53 RNA with the chemoenzymatic synthesis 15
   - 3.3 Preparation of the 2'dGsw^{86}-C26 RNA with a splinted ligation 17
   - 3.4 Synthesis and quality control of the 2'dGsw^{70}-A25 RNA 24
   - 3.5 Preparation of the 2'dGsw^{86}-C75-(5-D, 1',6-^{13}C) RNA with a splinted ligation 27
4. Solid state MAS-DNP measurements 30
   - 4.1 MAS DNP NMR experiments 30
   - 4.2 MAS-DNP TEDOR-analysis 31
   - 4.3 NMR spectra 34
5. Solution NMR binding model 37
6. References 37

Experimental Section

1. DNA and RNA sequences of the 2’dGsw^{86}-C26/C53/C75 and 2’dGsw^{70}-A25

All investigated RNAs carry a site-specifically isotope labeled modification in the aptamer domain of the deoxyguanosine-sensing riboswitch (2’dGsw) from *Mesoplasma florum*. Three constructs had a length of 86 nucleotides (2’dGsw^{86}-C26/C53/C75). These RNAs were produced by the chemoenzymatic synthesis or splinted ligation. The 2’dGsw^{70}-A25 construct with 70 nucleotides was synthesized by solid-phase chemical synthesis by the company *Innotope*. The secondary structure of the RNAs is shown in Figure S1. For sample 3 (2’dGsw^{86}-C26), the cytidine in position 20 was swapped with guanosine in position 81. This swap within the P1 stem of the target aptamer domain does not affect its secondary structure, but incorporation of this swap allows preparation of the sample in a single splinted ligation step, resulting in increased yields. The 2’dGsw^{70}-A25 RNA has a shortened and stabilized P1 stem for synthesis yield in the linear solid-phase chemical synthesis.

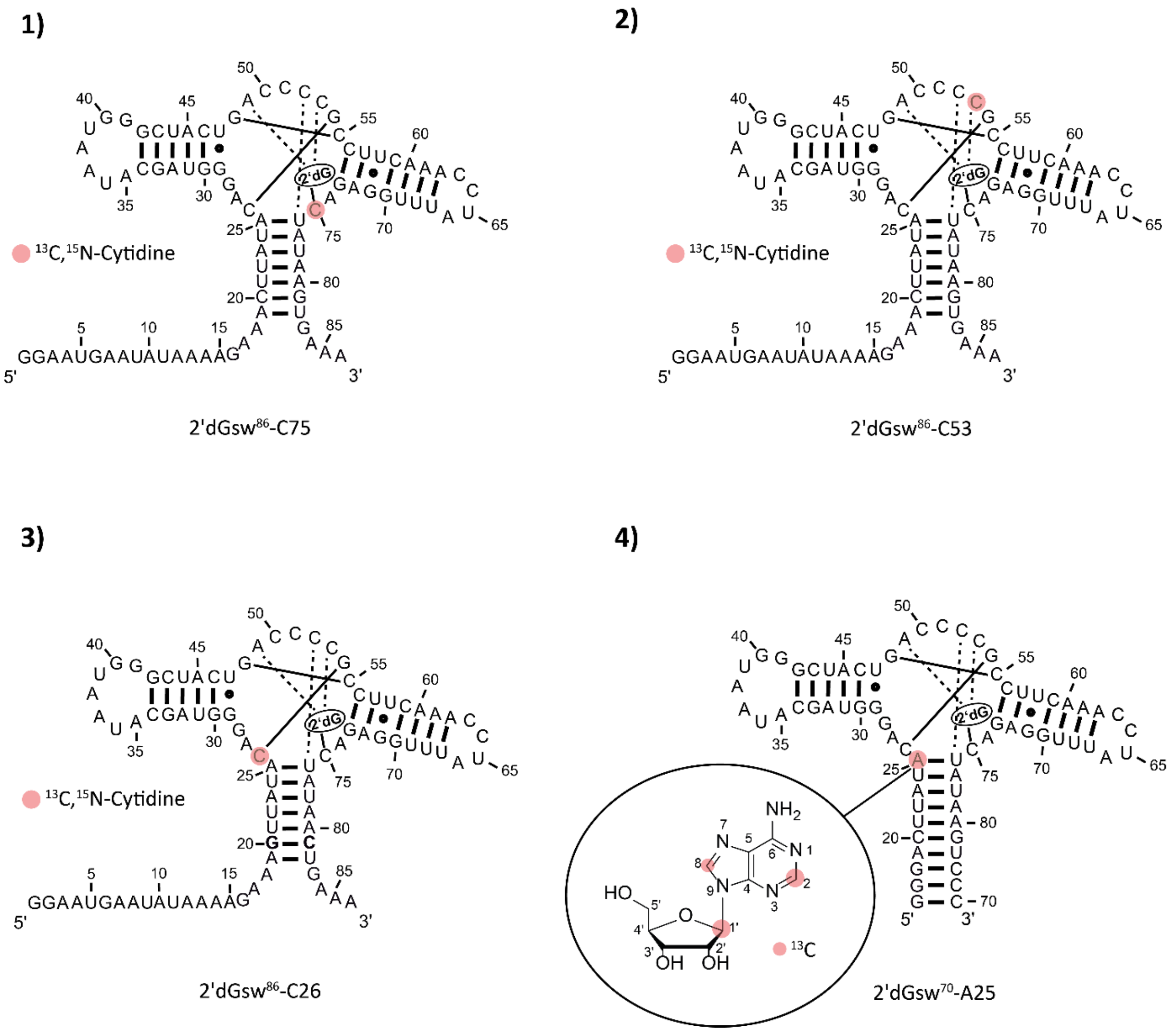

Figure S1 ***1)*** *&* ***2)*** *The secondary structures of the 2’dGsw^{86}-C75 and -C53 RNAs with a single ^{13}C,^{15}N-cytidine at position 75 or 53 (aptamer domain) are shown.* ***3)*** *The secondary structure of 2’dGsw^{86}-C26 is shown with a single ^{13}C,^{15}N-Cytidine at position 26. In contrast to the native sequence, the cytidine in position 20 is swapped with the guanosine in position 81 highlighted in bold.* ***4)*** *Shows the 2’dGsw^{70}-A25 RNA with a 1`,2,8 -^{13}C-labeled adenosine at position 25.*

DNA and RNA sequences used for the preparation of the desired constructs are listed in the following tables (Table S1 - Table S5).

2’dGsw^{86}-C75

Table S1 DNA and RNA sequences used for the preparation of the 2’dGsw^{86}-C75 RNA with the chemoenzymatic synthesis. The 2’dGsw$^{76-86}$ was purchased from Dharmacon. The 2’dGsw^{74} RNA was prepared via in vitro transcription (ivt). A DNA template from a PCR was used for the ivt. Square brackets at the reverse primer describe a methoxy modification at the 2' position. Use of 2’-methoxy primers improves RNA synthesis homogeneity during ivt.[1] “p” describes a phosphate group, which is necessary for the splinted ligation.

Name	Length	Sequence
2’dGsw^{74}	74mer RNA	5’-GGA AUG AAU AUA AAA GAA ACU UAU ACA GGG UAG CAU AAU GGG CUA CUG ACC CCG CCU UCA AAC CUA UUU GGA GA-3’
2’dGsw$^{76-86}$	11mer RNA	5’-p-UAU AAG UGA AA-3’
Long DNA Splint	84mer DNA Splint	5’-TTT CAC TTA TAG TCT CCA AAT AGG TTT GAA GGC GGG GTC AGT AGC CCA TTA TGC TAC CCT GTA TAA GTT TCT TTT ATA TTC ATT-3’
Fwd_dGsw^{74}	44mer DNA Primer	5'-TAA TAC GAC TCA CTA TAG GAA TGA ATA TAA AAG AAA CTT ATA CA-3'
Rev_dGsw^{74}	18mer DNA Primer	5’-[2’OMeU][2’OMeC]T CCA AAT AGG TTT GAA-3’

2’dGsw^{86}-C53

Table S2 DNA and RNA sequences used for the preparation of the 2’dGsw^{86}-C53 RNA. The 2’dGsw$^{54-86}$ RNA was purchased from Dharmacon. The 2’dGsw^{52} RNA was prepared via in vitro transcription (ivt). A DNA template from a PCR was used for the ivt. Square brackets at the reverse primer describe a methoxy modification at the 2′ position.

Name	Length	Sequence
2’dGsw^{52}	52mer RNA	5’-GGA AUG AAU AUA AAA GAA ACU UAU ACA GGG UAG CAU AAU GGG CUA CUG ACC C-3’
2’dGsw$^{54-86}$	33mer RNA	5’-p-GCC UUC AAA CCU AUU UGG AGA CUA UAA GUG AAA-3’
Long DNA Splint	84mer DNA Splint	5’-TTT CAC TTA TAG TCT CCA AAT AGG TTT GAA GGC GGG GTC AGT AGC CCA TTA TGC TAC CCT GTA TAA GTT TCT TTT ATA TTC ATT-3’
Fwd_dGsw^{52}	44mer DNA Primer	5’-TAA TAC GAC TCA CTA TAG GAA TGA ATA TAA AAG AAA CTT ATA CA-3’
Rev_dGsw^{52}	18mer DNA Primer	5’-[2’OMeG][2’OMeG]G TCA GTA GCC CAT TAT-3’

2’dGsw^{86}-C26

Table S3 DNA and RNA sequences used for the preparation of the 2’dGsw^{86}-C26 RNA with a splinted ligation. The 2’dGsw^{27} and 2’dGsw$^{28-86}$ RNAs were prepared via in vitro transcription (ivt). The ^{13}C,^{15}N-labeled nucleotide is highlighted in yellow. The sequence, which differs from the native structure of the 2’dGsw^{86} is highlighted in pink. Commercially purchased Fwd and Rev DNA were used as a template for the ivt. Square brackets at the reverse primer describe a methoxy modification at the 2′ position. “p” describes a phosphate group, which is necessary for the splinted ligation.

Name	Length	Sequence
2’dGsw^{27}	27mer RNA	5’-GGA AUG AAU AUA AAA GAA A**G**U UAU A**C**A-3’
2’dGsw$^{28-86}$	59mer RNA	5’-p-GGG UAG CAU AAU GGG CUA CUG ACC CCG CCU UCA AAC CUA UUU GGA GAC UAU AA**C** UGA AA-3’
Long DNA Splint	84mer DNA Splint	5’-TTT CAC TTA TAG TCT CCA AAT AGG TTT GAA GGC GGG GTC AGT AGC CCA TTA TGC TAC CCT GTA TAA GTT TCT TTT ATA TTC ATT-3’
Fwd_dGsw^{27}/ Fwd_dGsw$^{28-86}$	18mer DNA	5’-TAA TAC GAC TCA CTA TA G-3’
Rev_dGsw^{27}	44mer DNA	5’-[2OMeT][2OMeG]T ATA A**C**T TTC TTT TAT ATT CAT TCC TAT AGT GAG TCG TAT TA-3’
Rev_dGsw$^{28-86}$	76mer DNA	5’-[2OMeU][2OMeU]T CA**G** TTA TAG TCT CCA AAT AGG TTT GAA GGC GGG GTC AGT AGC CCA TTA TGC TAC CCT ATA GTG AGT CGT ATT A -3’

2’dGsw^{70}-A25

Table S4 RNA sequence of 2’dGsw^{70}-A25 RNA. The labeled nucleotide is highlighted in yellow. The RNA was produced by solid-phase chemical synthesis.

Name	Length	Sequence
2’dGsw^{70}-A25	70mer RNA	5`-GGG ACU UAU [(1’,2,8)$^{13}$C-**A**]CA GGG UAG CAU AAU GGG CUA CUG ACC CCG CCU UCA AAC CUA UUU GGA GAC UAU AAG UCC C-3`

2’dGsw^{86}-C75-(5-D, 1’,6-^{13}C)

Table S5 DNA and RNA Sequences used for the preparation of the 2’dGsw^{86}-C75-(5-D, 1’,6-^{13}C) RNA with a splinted ligation. The 2’dGsw^{74} RNA was prepared via ivt. The 2’dGsw$^{76-86}$ RNA was purchased from Innotope. The labeled nucleotide is highlighted in yellow. A DNA template from a PCR was used for the ivt. Square brackets at the reverse primer describe a methoxy modification at the 2′ position. “p” describes a phosphate group, which is necessary for the splinted ligation.

Name	Length	Sequence
2’dGsw^{74}	74mer RNA	5’-GGA AUG AAU AUA AAA GAA ACU UAU ACA GGG UAG CAU AAU GGG CUA CUG ACC CCG CCU UCA AAC CUA UUU GGA GA-3’
2’dGsw$^{75-86}$	12mer RNA	5’-p-[5-D, (1’,6)^{13}C-**C**]UA UAA GUG AAA -3’
Short DNA Splint	42mer DNA Splint	5’-TTT CAC TTA TAG TCT CCA AAT AGG TTT GAA GGC GGG GTC AGT-3’
Fwd_dGsw^{74}	44mer DNA Primer	5’-TAA TAC GAC TCA CTA TAG GAA TGA ATA TAA AAG AAA CTT ATA CA-3’
Rev_dGsw^{74}	18mer DNA Primer	5’-[2’OMeU][2’OMeC]T CCA AAT AGG TTT GAA-3’

Extinction coefficients

Table S6 Extinction coefficients which were used to calculate the concentration of the RNAs and DNAs. The values were taken from the vendor’s data sheet or calculated with an online tool[2].

Name	Extinction coefficient at 260 nm [mM^{-1} cm^{-1}]
2’dGsw^{74} RNA	876.5[2]
2’dGsw$^{76\text{-}86}$ RNA	127.0
2’dGsw^{52} RNA	633.1[2]
2’dGsw$^{54\text{-}86}$ RNA	341.4
2’dGsw^{27}	356.8[2]
2’dGsw$^{28\text{-}86}$ RNA	696.2[2]
2’dGsw^{70}-A25	782.9[2]
Long DNA Splint (84mer)	801.4
Short DNA Splint (42mer)	470.0[2]

2. Synthesis of the $^{13}C,^{15}N$-cytidine 3',5'-bisphosphate

2.1 General working techniques

The synthesis was performed under N_2 atmosphere. The starting material ($^{13}C,^{15}N$-cytidine) for the synthesis was purchased from *Silantes*. The purity of the starting material was confirmed by NMR spectroscopy and mass spectrometry. Diphosphoryl chloride and triethylamine were purchased from *Sigma-Aldrich®* (*Merck*) and *VWR™ Chemicals*, respectively, and used without further purification. Cooling of the reaction solution was performed with a Julabo FT902 immersion cooler. After reaction completion, the solvent was concentrated with the vacuum concentrator plus from Eppendorf. For freeze-drying, an Alpha 2-4 from Christ was used.

2.2 Synthesis of the $^{13}C,^{15}N$-cytidine 3',5'-bisphosphate

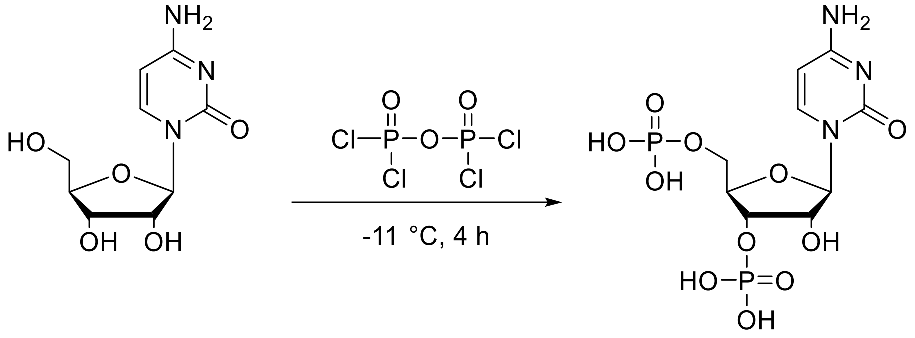

Figure S2 Reaction scheme for the 3',5'-bisphosphorylation of the $^{13}C,^{15}N$-cytidine with diphosphoryl chloride under a protective atmosphere.

15 mg (0.059 mmol, 1 eq.) of $^{13}C,^{15}N$-cytidine were cooled to a temperature of -11 °C. 0.298 mL (2.057 mmol, 35 eq.) of diphosphoryl chloride were added and the reaction mixture was stirred for 4 h at the previously set temperature. After complete conversion (thin-layer chromatography (TLC) control), excess diphosphoryl chloride was hydrolyzed by adding ice. The pH value was adjusted to 6.5 with 1 M TEAB buffer (1 M triethylammonium bicarbonate, pH 8). The solvent was removed through a vacuum concentrator at 4 °C and the product was purified by RP-HPLC, see Figure S3 (Column: MZ Aqua Perfect 100 Å, 5 µm, [4.6x250 mm analytical], [10x250 mm preparative], gradient see Table S7). The purified product was freeze-dried, then resolved in 10 mL water and lyophilized again. This process of co-evaporation was repeated ten times to remove the RP-HPLC buffer. The yield was determined using the NanoDrop™ ND-1000 spectrophotometer from Thermo Scientific. An extinction coefficient of 9000 $M^{-1}cm^{-1}$ at 271 nm was used for the determination of the yield.[3]

Table S7 RP-HPLC gradient for the purification of the $^{13}C,^{15}N$-cytidine 3',5'-bisphosphate at room temperature. 0.4 M triethylammonium bicarbonate (TEAB) with pH 8 and acetonitrile were used as RP-HPLC buffers.

Time [min]	0.4 M TEAB [%]	Acetonitrile [%]
0	100	0
10	100	0
40	70	30
50	50	50

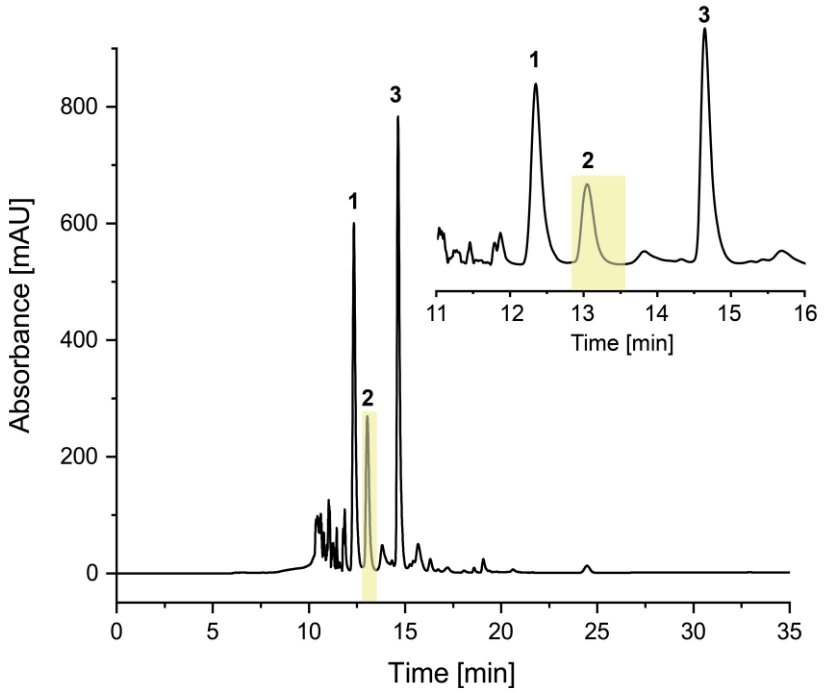

Figure S3 RP-HPLC chromatogram of the ^{13}C,^{15}N-cytidine 3',5'-bisphosphate purification. The product has a retention time of ca. 13 min and is highlighted in yellow. The remaining signals are caused by by-products such as monophosphates.

2.3 Characterization of synthesized ^{13}C,^{15}N-cytidine 3',5'-bisphosphate

The product was characterized by high resolution mass spectrometry (HRMS) with a MicrOTOF from Bruker and by NMR spectroscopy with a 500 MHz Bruker spectrometer (AV500) and with a 5 mm Prodigy BBO probe head at 298 K.

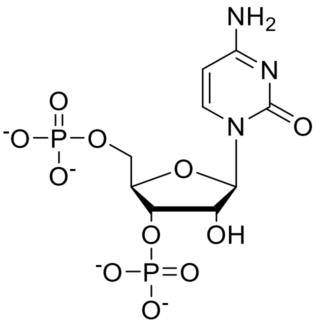

p(^{13}C,^{15}N-C)p

Yield: 2.2 mg, (9% of the ^{13}C,^{15}N-cytidine 3',5'-bisphosphate)

HRMS (ESI): m/z calculated for $^{13}C_9H_{15}{}^{15}N_3O_{11}P_2$ + H^+ 416.04676 [M+H$^+$]; found: 416.0468 (Δm= 0.00004, error 0.1ppm).

^{1}H-NMR (^{13}C-decoupled) (D_2O, 500 MHZ): δ [ppm]= 8.06 (dd, 1H, ^3J(H,H)=7.0 Hz, ^2J(H,N)=29.0 Hz, H-6), 6.16 (s, 1H, H-5), 4.70 (m, 1H, H-3'), 4.47 (s, 1H, H-1'), 4.43 (s, 1H, H-2'), 4.30 (s, 1H, H-4'), 4.11-4.06 (m, 2H, H-5')

Acquisition Parameter

Source Type	ESI	Ion Polarity	Positive	Set Nebulizer	1.0 Bar
Focus	Active	Set Capillary	4000 V	Set Dry Heater	220 °C
Scan Begin	100 m/z	Set End Plate Offset	-500 V	Set Dry Gas	5.0 l/min
Scan End	1000 m/z	Set Collision Cell RF	150.0 Vpp	Set Divert Valve	Waste

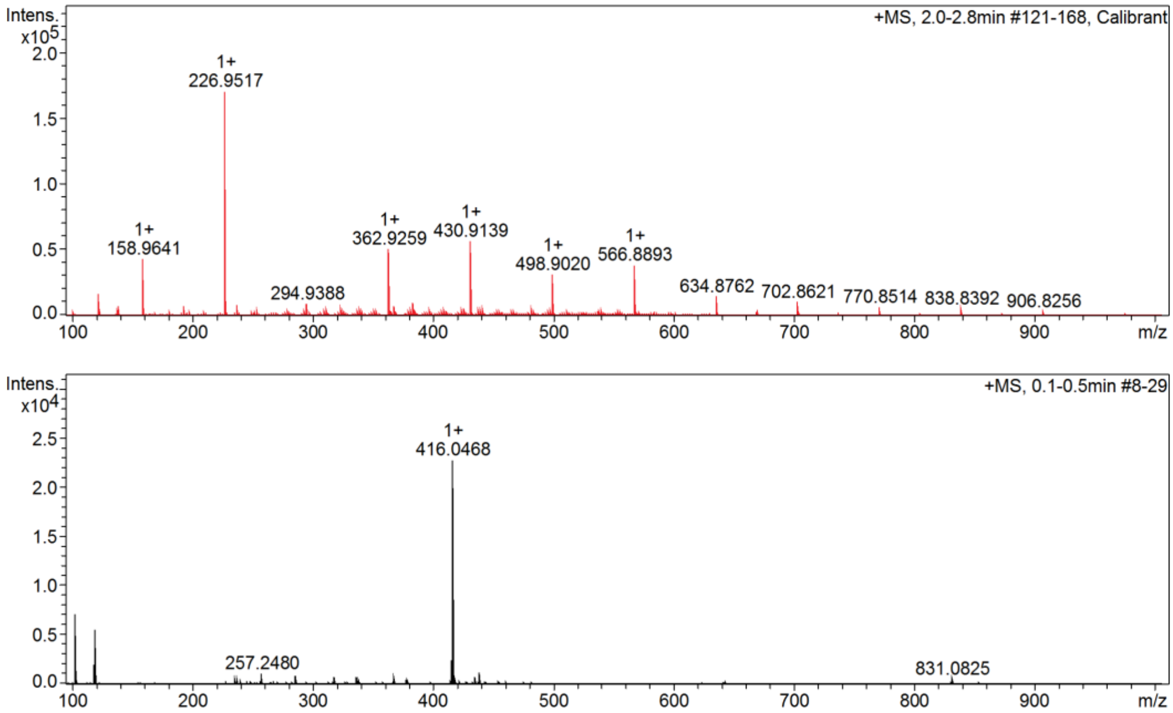

Figure S4 HRMS spectrum of the product ^{13}C,^{15}N-cytidine 3’,5’- bisphosphate after RP-HPLC purification.

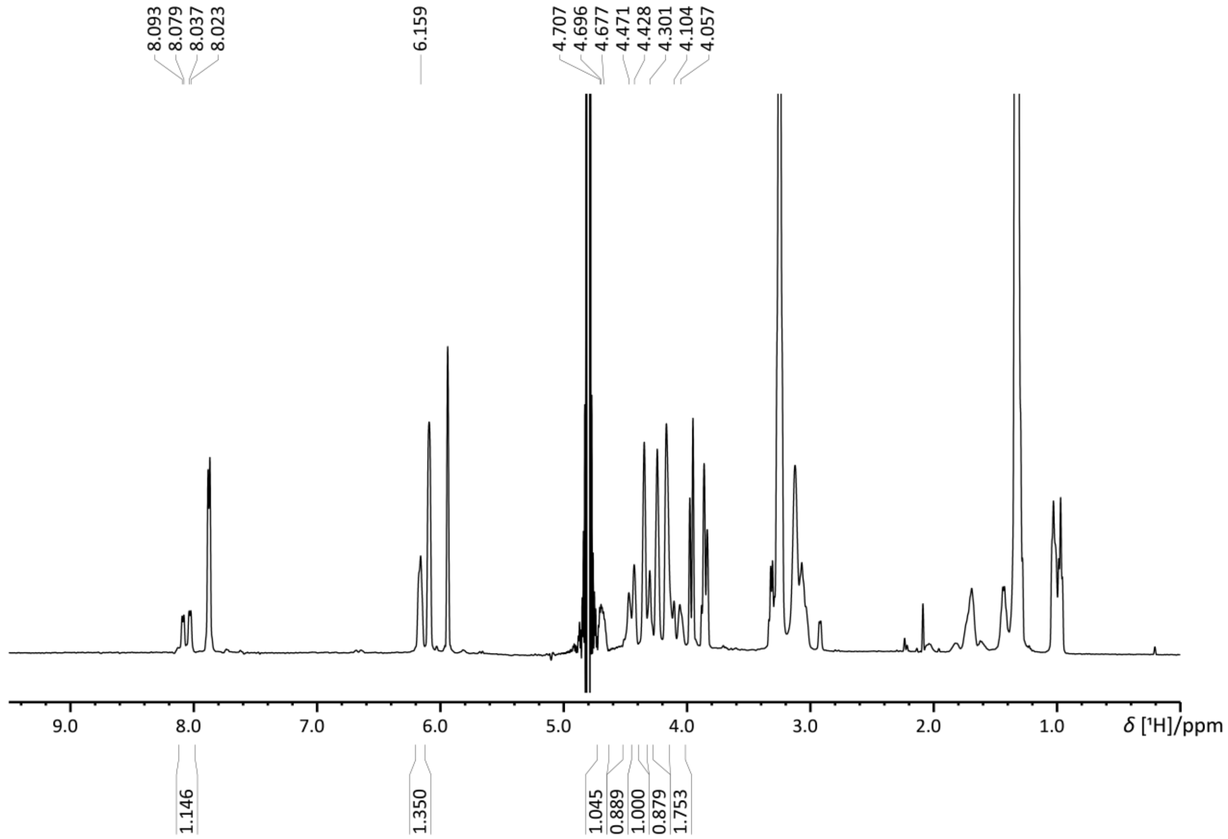

Figure S5 ^1H-1D NMR spectrum of p(^{13}C,^{15}N-C)p in D_2O (500 MHz, 298 K) after RP-HPLC purification. The integrals and chemical shifts are only shown for the product signals. The other signals arise from the starting material and the by-product ^{13}C,^{15}N-cytidine 2',5'-bisphosphate.

3. Preparation of the site-specific labeled RNAs 2’dGsw^{86}-C26/C53/C75 and 2’dGsw^{25}-A25

The 2’dGsw^{70}-A25 RNA was synthesized using solid-phase chemical synthesis at the company *Innotope*. The sample was delivered purified and could be used for the measurements without further purification. Sample preparation and quality control are described in chapter 3.4.

The 2’dGsw^{86}-C75 and 2’dGsw^{86}-C53 RNAs were prepared with the chemoenzymatic approach (see chapters 3.1 and 3.2). The 2’dGsw^{86}-C26 RNA was prepared using a single splinted ligation (see chapter 3.3). In case of single splinted ligation less enzymatic steps are required compared to the chemoenzymatic approach, which leads to a significant higher yield. However, this method is not always applicable and will depend on the position of the desired modification.[4]

3.1 Preparation of the 2’dGsw^{86}-C75 RNA with the chemoenzymatic synthesis

The chemoenzymatic synthesis has been described elsewhere [4,5]. In brief, it involves three enzymatic steps. In the 3’-extension the ^{13}C,^{15}N-cytidine 3’,5’-bisphosphate is ligated to the acceptor 2’dGsw^{74} RNA, followed by dephosphorylation to remove the 3’-end phosphate group. This is necessary for the last enzymatic step, the splinted ligation.

3.1.1 DNA template preparation by PCR for the 2’dGsw^{74} RNA

The DNA was amplified via a Polymerase Chain Reaction (PCR) from a plasmid containing the full sequence of the 2’dG-sw. The PCR primer sequences are shown in Table S1. Two nucleotides of the reverse PCR primer contain methoxy modifications at the 2’ position in the 5’-end, which provided homogeneity during transcription.[1] The DNA template includes a T7 promoter sequence at the 5’-end required for the T7 RNA polymerase (T7 RNAP) recognition. The PCR components are shown in Table S8. The volume of a reaction tube was 50 µL. 50 cycles were applied for the PCR. The corresponding temperatures are shown in Table S9. Several 50 µL batches were prepared to ensure a sufficient amount for the *in vitro* transcription.

Table S8 PCR reaction components for DNA amplification. The DNA was used without further purifications for the in vitro transcription of the 2’dGsw^{74} RNA.

	**PCR**
**Plasmid**	40 ng
**Fwd-primer**	5.0 µM
**Rev-primer**	5.0 µM
**NTPs**	200 µM
***Phusion® High-Fidelity* DNA buffer**	1x
**1 U/50 µL Phusion® DNA polymerase**	1% (v/v)
**ddH$_2$O**	Add up to 50 µL

Table S9 Thermocycler program for the PCR for the preparation of the 2'dGsw^{74} RNA. 50 cycles were used (step 2-4).

Step	Temperature [°C]	Time [s]
1	98	120
2	55	20
3	72	15
4	98	10
5	4	-

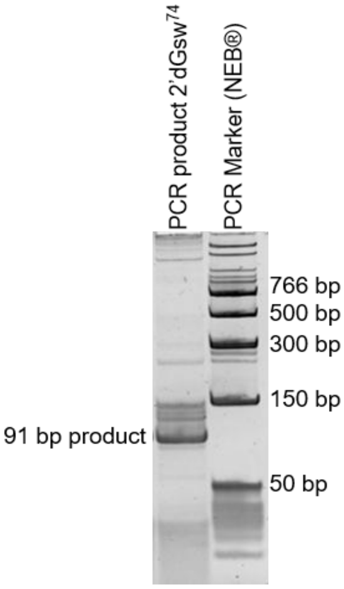

Figure S6 Native 10% polyacrylamide gel with PCR product (91 bp) of the DNA template for the 2'dGsw^{74} RNA. The running time was 40 min at 140 V. The gel was visualized via GelRed and Bio Rad ChemiDoc XRS+.

3.1.2 Preparative *in vitro* transcription of the 2'dGsw^{74} RNA and purification

The preparative *in vitro* transcription (ivt) was performed in a 10 mL reaction volume with 30 µg/mL homemade T7 RNA polymerase[6] in 1x transcription buffer (200 mM tris/glutamate pH 8.1) with 2 mM spermidine, 20 mM dithiothreitol (DTT) and 20% (v/v) DMSO. Optimized conditions with 40 mM $Mg(OAc)_2$, 800 µL unpurified PCR product and 10 mM rNTP (ATP 3.6 mM, CTP 1.9 mM, GTP 2.2 mM, UTP 2.3 mM) were used. The transcription reaction mixture was incubated for 1 h at 37 °C and 70 rpm. As soon as precipitation was visible, 80 µL of Inorganic Pyrophosphatase (YIPP) (100 U/mL, NEB®) was added and the batch was incubated for another 7 h at the same conditions. The 2'dGsw^{74} RNA was analyzed by polyacrylamide gel electrophoreses after the ivt (Figure S7). The purity was good. Only the desired RNA was obtained, no by-products were transcribed. The purification to remove buffer and rNTPs was performed by centrifugal concentrator (Vivaspin™ 20 from *Sartorius*, 5000 MWCO). A yield of 141 nmol was achieved.

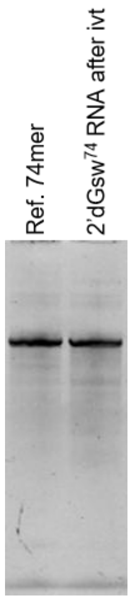

Figure S7 Denaturing polyacrylamide gel (15%) of the 2'dGsw^{74} RNA after ivt. The run time was 53 min at 180 V. The gel was visualized via GelRed and Bio Rad ChemiDoc XRS+.

3.1.3 3'-Extension of the 2'dGsw^{74} RNA with ^{13}C,^{15}N-cytidine 3',5'-bisphosphate

The purified 2'dGsw^{74} RNA was ligated with ^{13}C,^{15}N-cytidine 3',5'-bisphosphate using T4 Rnl1. Several batches with a volume of 100 µL each were prepared according to Table S10. The ligation reactions were incubated for 20 h at 300 rpm and 37 °C. In addition, two negative controls, one without T4 Rnl1 and the second without ^{13}C,^{15}N-cytidine 3',5'-bisphosphate, were prepared to verify whether the oxidation works. The negative controls were treated as all the other samples in the following procedure (chapters 3.1.3-3.1.6). After the incubation the enzyme was inactivated (15 min at 65 °C) and the 3'-extension buffer components were removed through buffer exchange with ddH_2O via a centrifugal concentrator (Vivaspin™ 20 from *Sartorius*, 5000 MWCO). Note that removal of the buffer is essential.[4]

Table S10 Composition of the 3'-Extension of the 2'dGsw^{74} RNA with the ^{13}C,^{15}N-cytidine 3',5'-bisphosphate under optimized conditions. Normally a 4-fold excess of the modified nucleoside 3',5'-bisphosphate is used. The synthesis of ^{13}C,^{15}N-cytidine 3',5'-bisphosphate showed by-products after purification that could not be removed. Therefore, a 12-fold excess of 600 µM was used.

	**Quantity**
2'dGsw^{74} RNA	50 µM
^{13}C,^{15}N-cytidine 3',5'-bisphosphate	600 µM
T4 RNA Ligase reaction buffer	1x
DMSO	20%
T4 RNA Ligase 1	5 µL (10 units per nmol of RNA)
ATP	1 mM
ddH_2O	Filled up to 100 µL

3.1.4 Oxidation of the non-ligated 2'dGsw^{74} RNA after the 3'-extension

The non-ligated 2'dGsw^{74} RNA was removed from the enzymatic pathway by oxidation with $NaIO_4$. 20 µM of the RNA were mixed with 40 mM $NaIO_4$ in a total volume of 150 µL and incubated for 2 h and 300 rpm at room temperature (25 °C) under exclusion of light. The reaction was quenched by adding 0.5 volumes of 50% (v/v) ethylene glycol and incubated for 5 min at 300 rpm. Afterwards a precipitation from ethanol was performed by adding sodium acetate solution (pH 5.5) to a final concentration of 0.3 M and the addition of 2.5 times of the sample volume of 100% ethanol (cold). The batches were incubated overnight (18 h) at -25 °C and then were centrifuged at 10000 g at -4 °C for 60 min. The RNA pellets were dried with a vacuum concentrator for 15 min and reconstituted in 250 µL ddH_2O. The salt was removed via buffer exchange with a centrifugal concentrator (Vivaspin™ 2 from *Sartorius*, 5000 MWCO).

3.1.5 Dephosphorylation of the 2'dGsw^{74}-p(^{13}C,^{15}N)Cp RNA

The phosphate group at the 3'-end of the 2'dGsw^{74}-p(^{13}C,^{15}N)C**p** RNA was removed by shrimp alkaline phosphatase (rSAP). 22 µM of RNA were mixed with 1x CutSmart® buffer and 2 units rSAP in a total volume of 50 µL. The dephosphorylation reaction mixture was incubated for 22 h at 37 °C and 300 rpm. Afterwards rSAP was deactivated by heating the mixture for 5 min at 65 °C. No further purification steps were applied.

3.1.6 Splinted ligation of the dephosphorylated 2'dGsw^{74}-p(^{13}C,^{15}N)C RNA with the 2'dGsw$^{76\text{-}86}$ 11mer RNA and NMR sample preparation

The 11mer donor 2'dGsw$^{76\text{-}86}$ RNA with a phosphate group at the 5'-end was ligated with the dephosphorylated 2'dGsw^{74}-p(^{13}C,^{15}N)C RNA by the T4 RNA Ligase 2(T4 Rnl2). The pipetting scheme for the splinted ligation is demonstrated in Table S11. 46 reaction mixtures (each 250 µL) were prepared in total for the NMR sample. Increasing the volume reduces the ligation efficiency.[4] The reaction mixtures were heated to 80 °C for 4 min without T4 Rnl2 and then cooled to 37 °C within 10 min. Then incubated for 15 min at 37 °C and 300 rpm. Then 1% (v/v) in-house produced T4 Rnl2 (2 mg/mL) was added and the mixtures were incubated for another 3 h at 37 °C and 300 rpm. Then a phenol/chloroform/isoamyl alcohol (PCI) extraction using the 5 Prime Phase Lock Gel from *Quantabio* was performed. Followed by precipitation from ethanol as described in the previous chapter 3.1.4. Three splinted ligation mixtures were combined by resolving in a total volume of 100 µL ddH_2O. The DNA splint was removed with Turbo™ DNase (*ThermoFischer*) by adding 11 µL 10x TURBO™ DNase Reaction Buffer, 6 U TURBO™ DNase and incubated at 37 °C for 3 h. Subsequently the RNA was purified by RP-HPLC. The RP-HPLC purification was performed at 60 °C with the column XBridge Peptide BEH C18 (300 Å, 3,5 µm, 4,6x250 mm) from Waters. RP-HPLC gradient is shown in Table S12. The RP-HPLC buffer was removed with a lyophilizer and the RNA pellets were reconstituted in ddH_2O. The fractions were analyzed by analytical denaturing PAGE and the fractions containing clean product were combined (Figure S8). The yield of the product 2'dGsw^{86}-C75 RNA was 14 nmol (10% starting from the 3'-Extension). 2 eq. of ^{13}C,^{15}N-2'dG ligand and 40 eq. of $MgCl_2$ were added to the combined product fractions. Then ddH_2O was removed in a vacuum concentrator and the sample was reconstituted in 15 µL NMR-Buffer (50 mM KCl, 25 mM K_2HPO_4/KH_2PO_4 at pH 6.2). 15 µL d8-glycerol and 0.3 µmol AsymPolPOK were added to yield a final total concentration of 10 mM AsymPolPOK. The solution was transferred to a 3.2 mm Sapphire MAS NMR rotor and a small Teflon insert was added before closing the rotor.

Table S11 Composition of the splinted ligation of the dephosphorylated 75mer 2'dGsw^{74}-p(^{13}C,^{15}N)C RNA with the 11mer 2'dGsw$^{76\text{-}86}$ RNA.

	Quantity
Dephosphorylated 2'dGsw^{74}-p(^{13}C,^{15}N)C RNA	4.4 µM
11mer 2'dGsw$^{76\text{-}86}$ RNA	4.8 µM
84mer long DNA Splint	4.4 µM
T4 DNA Ligase buffer	1x
ddH_2O	Filled up to 250 µL
In house produced T4 RNA Ligase 2	1% (v/v) added after annealing

Table S12 RP-HPLC Gradient for the purification of 2'dGsw^{86}-C75 RNA after splinted ligation and DNA splint digestion. Hexafluoro-2-propanol (HFIP) buffer was prepared with 0.4 M HFIP and 16.3 mM triethylamine (TEA) in ddH_2O. XBridge Peptide BEH C18 (300 Å, 3,5 µm, 4,6x250 mm) column from Waters was used. The RP-HPLC was performed at 60 °C.

Time	0.4 M HFIP buffer (%)	Methanol (%)
0	95	5
3	95	5
8	90	10
35	55	45
43	0	100

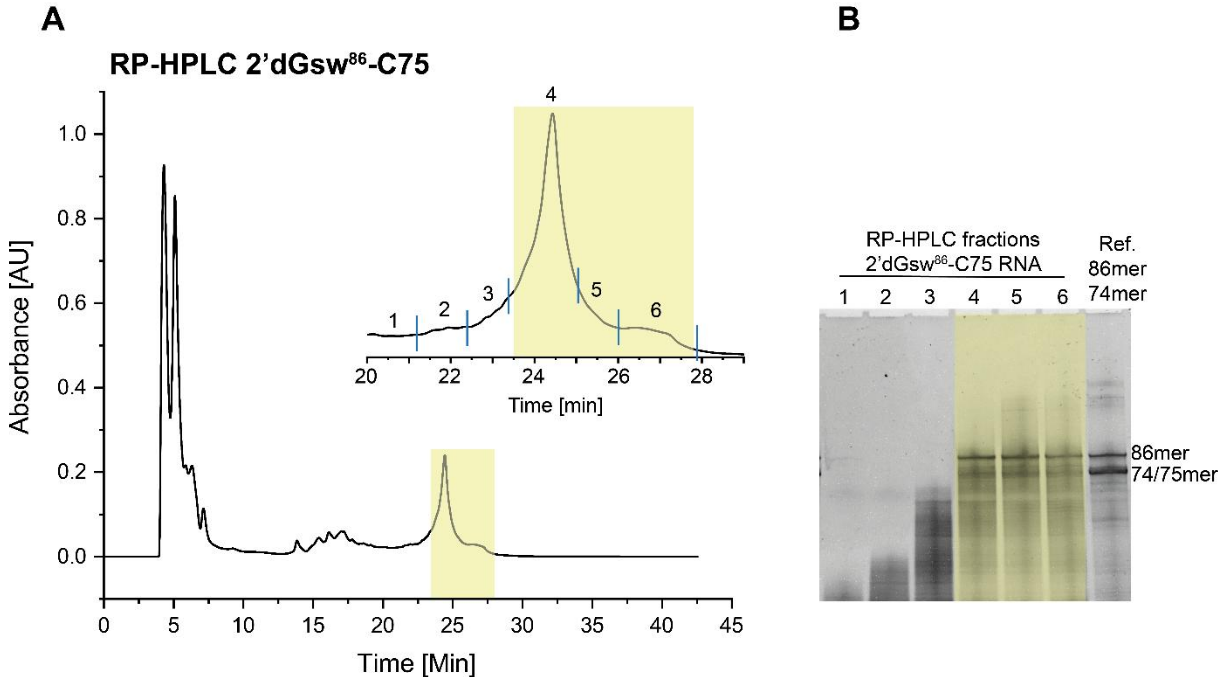

*Figure S8 **A**) RP-HPLC chromatogram of the 2'dGsw^{86}-C75 RNA purification. The product RNA is highlighted in yellow and has a retention time of 23.5-28.0 min. **B**) Denaturing polyacrylamide gel (10%) of the RP-HPLC purification of the 2'dGsw^{86}-C75 RNA. Product fractions are highlighted in yellow. The run time was 29 min at 260 V.*

3.2 Preparation of the 2’dGsw^{86}-C53 RNA with the chemoenzymatic synthesis

3.2.1 DNA template preparation by PCR for the 2’dGsw^{52} RNA

The DNA template was prepared as described in chapter 3.1.1 via a PCR from a plasmid containing the full sequence of the 2’dG-sw. The PCR primer sequences are demonstrated in Table S2.

3.2.2 Preparative *in vitro* transcription of the 2’dGsw^{52} RNA and purification

The preparative ivt was performed in a 10 mL reaction volume with 30 µg/mL homemade T7 RNA polymerase[6] in 1x transcription buffer (200 mM tris/glutamate pH 8.1) with 2 mM spermidine, 20 mM DTT and 20% (v/v) DMSO. Optimized conditions with 10 mM $Mg(OAc)_2$, 800 µL unpurified PCR product and 10 mM rNTP (ATP 4.0 mM, CTP 1.5 mM, GTP 2.3 mM, UTP 2.1 mM) were used. The transcription reaction mixture was incubated for 1 h at 37 °C and 70 rpm. As soon as precipitation was visible, 80 µL of YIPP (100 U/mL, NEB®) was added and the batch was incubated for another 5 h at the same conditions. The 2’dGsw^{52} RNA was analyzed by polyacrylamide gel electrophoreses after the ivt (Figure S9). The desired product RNA has predominantly formed. However, by-product bands with significantly weaker intensity above the target RNA are visible. The purification to remove buffer and rNTPs was performed by centrifugal concentrator (Vivaspin™ 20 from *Sartorius*, 5000 MWCO). A yield of 370 nmol was achieved.

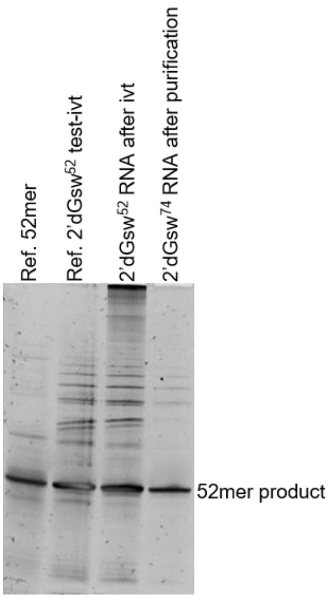

Figure S9 Denaturing polyacrylamide gel (12%) of the 2’dGsw^{52} RNA after ivt and purification with a centrifugal concentrator. The run time was 80 min at 140 V. The gel was visualized via GelRed and Bio Rad ChemiDoc XRS+.

3.2.3 3’-Extension of the 2’dGsw^{52} RNA with ^{13}C,^{15}N-cytidine 3’,5’-bisphosphate, oxidation and dephosphorylation

The 3’-extension of the 2’dGsw^{52} RNA was performed as described in chapter 3.1.3. The subsequent oxidation and dephosphorylation of the ligated 2’dGsw^{52}-p(^{13}C,^{15}N)Cp RNA were performed as described in chapters 3.1.4 and 3.1.5.

3.2.4 Splinted ligation of the dephosphorylated 2’dGsw^{52}-p(^{13}C,^{15}N)C RNA with the 2’dGsw$^{54\text{-}86}$ 33mer RNA and NMR sample preparation

The 33mer donor 2’dGsw$^{54\text{-}86}$ RNA with a phosphate group at the 5’-end was ligated with the dephosphorylated 2’dGsw^{52}-p(^{13}C,^{15}N)C RNA by the T4 RNA Ligase 2(T4 Rnl2) under optimized conditions.[4] The pipetting scheme for the splinted ligation is demonstrated in Table S13. 52 reaction mixtures (each 250 µL) were prepared in total for the NMR sample. Increasing the volume reduces the ligation efficiency.[4] The reaction mixtures were heated to 80 °C for 4 min without T4 Rnl2 and then cooled to 37 °C within 10 min. The mixtures were incubated for 15 min at 37 °C and 300 rpm. Then 1% (v/v) in-house produced T4 Rnl2 (2 mg/mL) was added, and the mixtures were incubated for another 3 h at 37 °C and 300 rpm. Then a phenol/chloroform/isoamyl alcohol (PCI) extraction using the 5 Prime Phase Lock Gel from *Quantabio* was performed. Followed by a precipitation from ethanol as described in chapter 3.1.5. The DNA splint was removed with Turbo™ DNase (*ThermoFischer*) as described in the chapter 3.1.6. Subsequently the RNA was purified by RP-HPLC. The RP-HPLC purification was performed at 60 °C with the column XBridge Peptide BEH C18 (300 Å, 3,5 µm, 4,6x250 mm) from Waters. RP-HPLC gradient is shown in Table S12. The RP-HPLC buffer was removed with a lyophilizer and the RNA pellets were reconstituted in ddH_2O. The fractions were analyzed by analytical denaturing PAGE and the fractions containing clean product were combined (Figure S10). The yield of the product 2’dGsw^{86}-C53 RNA was 11.5 nmol (5% starting from the 3’-Extension). 2 eq. of ^{13}C,^{15}N-2’dG ligand and 40 eq. of $MgCl_2$ were added to the combined product fractions. Afterwards the RNA was folded by heating the sample to 80 °C for 30 sec and then placing it on ice for 10 minutes. Then ddH_2O was removed in a vacuum concentrator and reconstituted in 15 µL NMR-Buffer (50 mM KCl, 25 mM K_2HPO_4/KH_2PO_4 at pH 6.2). 15 µL d8-glycerol and 0.3 µmol AsymPolPOK were added to yield a final total concentration of 10 mM AsymPolPOK. The solution was transferred to a 3.2 mm Sapphire MAS NMR rotor and a small Teflon insert was added before closing the rotor.

Table S13 Pipetting scheme for the splinted ligation of the dephosphorylated 2’dGsw^{52}-p(^{13}C,^{15}N)C RNA with the 33mer donor 2’dGsw$^{54\text{-}86}$ RNA.

	**Quantity**
Dephosphorylated 2’dGsw^{52}-p(^{13}C,^{15}N)C RNA	4.4 µM
33mer 2’dGsw$^{54\text{-}86}$ RNA	4.0 µM
84mer long DNA Splint	4.0 µM
T4 DNA Ligase buffer	1x
ddH_2O	Filled up to 250 µL
In house produced T4 RNA Ligase 2	1% (v/v) added after annealing

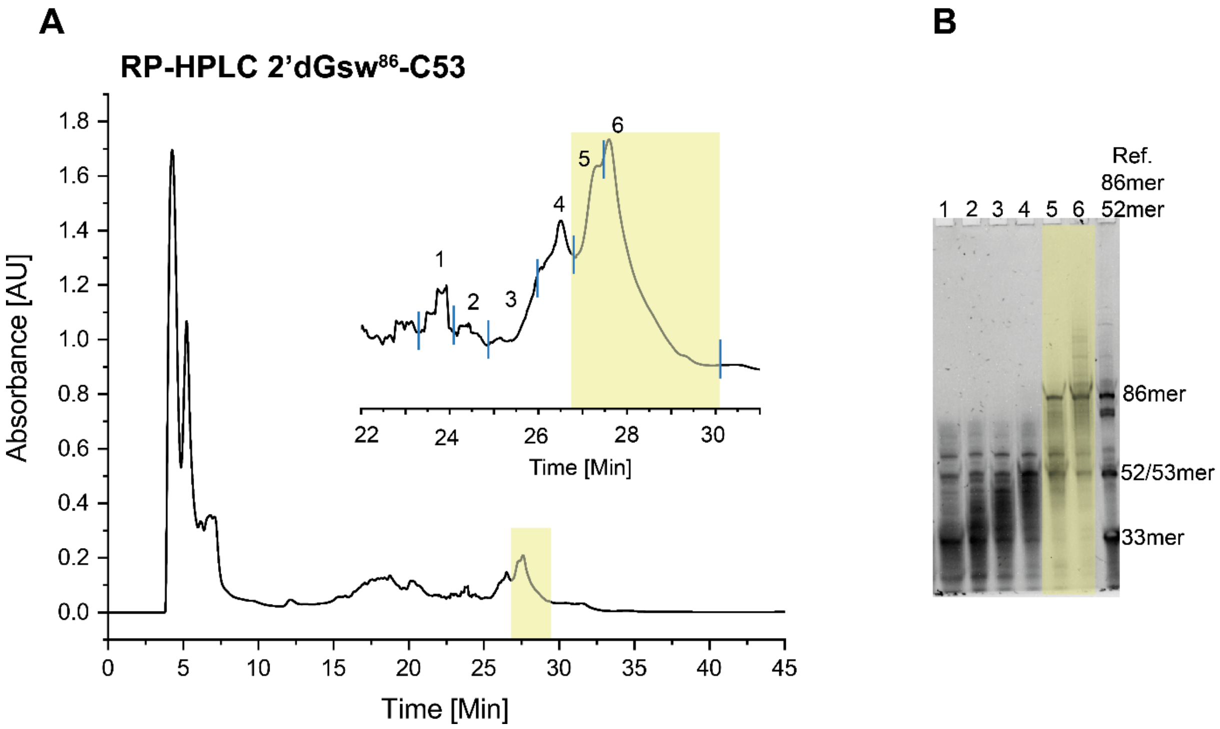

Figure S10 ***A****) RP-HPLC chromatogram of the 2'dGsw*^{86}*-C53 RNA purification. The product RNA is highlighted in yellow and has a retention time of ca. 27.0-30.0 min.* ***B****) Denaturing polyacrylamide gel (10%) of the RP-HPLC purification of the 2'dGsw*^{86}*-C53 RNA. The band below the 86mer reference is a degradation by-product of the 86mer. The run time was 45 min at 180 V.*

3.3 Preparation of the 2'dGsw^{86}-C26 RNA with a splinted ligation

For the preparation of the 2'dGsw^{86}-C26 RNA the cytidine in position 20 and guanosine in position 81 were swapped compared to the native structure. This allows for the preparation of the 2'dGsw^{86}-C26 RNA with only one enzymatic step, the splinted ligation. This speeds up the synthesis and increases the yield significantly compared to the chemoenzymatic synthesis.

For this purpose, the 2'dGsw^{86} was divided into two sections, the 27mer 2'dGsw^{27} and 59mer 2'dGsw$^{28-86}$ RNAs. Through the change of cytidine and guanosine, the 27mer 2'dGsw^{27} RNA has only one cytidine in the sequence at position 26, see Figure S1. When ^{13}C,^{15}N-cytidine triphosphate is added to the ivt of the 27mer 2'dGsw^{27} RNA, only the cytidine at position 26 will be isotopically labeled. Subsequently, the 27mer 2'dGsw^{27} RNA was ligated with the 59mer 2'dGsw$^{28-86}$ RNA in a splinted ligation and thus the desired 86mer 2'dGsw^{86}-C26 RNA with a ^{13}C,^{15}N-cytidine at position 26 was obtained.

3.3.1 In vitro transcription optimization of the 2'dGsw^{27} RNA

As DNA templates for the ivt of the 27mer 2'dGsw^{27} RNA, commercially purchased (*Eurofins*) forward (Fwd) and reverse (Rev) DNAs were used. The Rev DNA has the complete sequence of the RNA to be transcribed and the T7 promotor sequence. The Fwd DNA has only the T7 promoter sequence. The sequences of the Rev and Fwd DNAs are demonstrated in Table S3.

The Rev/Fwd DNA template concentration was optimized. Therefore, a test *in vitro* transcription in a total volume of 50 µL with different Rev/Fwd DNA concentrations was performed (0.25/0.5/1/2/4/6 pmol). For the test ivt 1x transcription buffer (200 mM tris/glutamate pH 8.1) with

2 mM spermidine, 20 mM DTT, 20% (v/v) DMSO, 10 mM $Mg(OAc)_2$ and 5 mM rNTP mix (1.25 mM each rNTP) were used. The transcriptions were incubated without the rNTPs and enzymes for 4 min and 80 °C and cooled within 10 min to 37 °C. Then the transcription mixture was incubated for 15 min at 37 °C and 300 rpm. Preheated rNTPs (37 °C, 15 min) was added. After that T7 RNA polymerase and YIPP (0.1 U/µL, NEB®) were added and the mixture was incubated for 3 h at 37 °C. The results were analyzed by polyacrylamide gel electrophoreses (Figure S11). The best DNA template condition were 2 and 6 pmol in a 50 µL reaction volume.

Subsequently the magnesium optimization with the optimized DNA template conditions (2&6 pmol) was performed as described in the previous paragraph. A range between 5 – 50 mM $Mg(OAc)_2$ was tested. 35 mM $Mg(OAc)_2$ and 6 pmol DNA template (in a 50 µL reaction volume) were chosen as the optimal conditions for the preparative ivt.

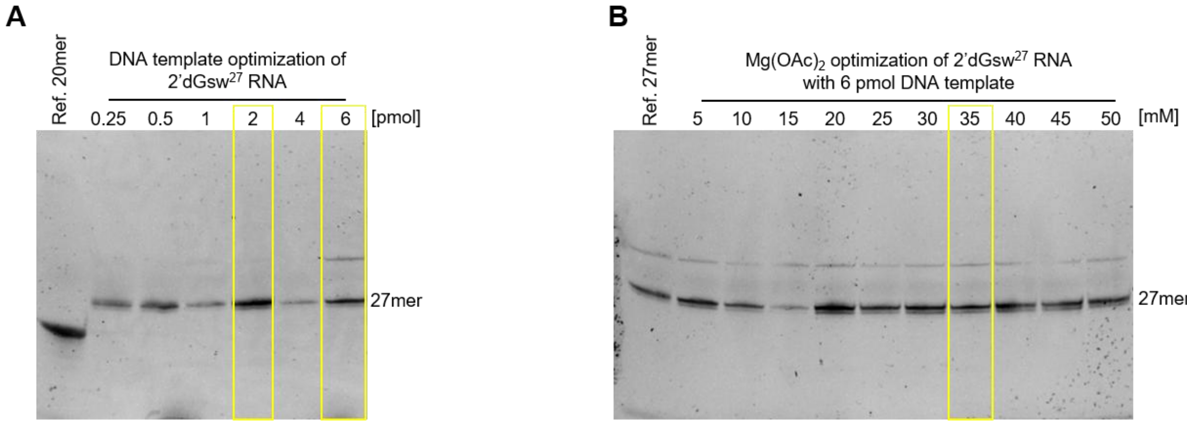

*Figure S11 Denaturing polyacrylamide gels (20%) of **A**) the DNA template optimization for the 2'dGsw[27] RNA. 0.25 – 6 pmol were tested. **B**) $Mg(OAc)_2$ optimization with the optimized DNA template conditions (6 pmol). The optimal conditions are highlighted in yellow. 35 $Mg(OAc)_2$ and 6 pmol were chosen as optimal conditions for the preparative in vitro transcription. The running time for both polyacrylamide gels was 50 min at 230 V. The gels was visualized via GelRed and Bio Rad ChemiDoc XRS+.*

3.3.2 Preparative *in vitro* transcription of the 2'dGsw[27] RNA and purification

The preparative ivt of the 27mer 2'dGsw[27] RNA was prepared in a 10 mL reaction volume with optimized DNA template and $Mg(OAc)_2$ conditions according to Table S14. The mixture was incubated without rNTPs and enzyme for 10 minutes at 80 °C and cooled to 37 °C within 80 minutes to anneal the Ref/Fwd DNA templates. After that the mixture was incubated at 37 °C and 70 rpm for 15 min. Then rNTPs were added and the mixture was incubated at the same conditions for further 15 min. The transcription was started with the addition of T7 RNA polymerase. The incubation time was 6.5 h and 70 rpm at 37 °C. 2 h after the start of transcription, YIPP was added. After the transcription buffer and remaining rNTP were removed with a centrifugal concentrator (Vivaspin™ 20 from *Sartorius*, 3000 MWCO). The RNA was analyzed by polyacrylamide gel electrophoreses (Figure S12). The desired RNA with a yield of 158.4 nmol was obtained.

Table S14 Composition for the preparative ivt of 27mer 2'dGsw[27] RNA under optimized conditions. Nat. ab. means natural abundance.

	**Quantity**
Transcription buffer (200 mM tris/glutamate pH 8.1)	200 mM
DTT	20 mM
Spermidine	2 mM
$Mg(OAc)_2$	35 mM
DMSO	20%
ATP nat. ab.	5.6 mM
CTP ^{13}C, ^{15}N	0.4 mM
GTP nat. ab.	1.9 mM
UTP nat. ab	2.2 mM
Fwd DNA template (Fwd_dGsw[27])	1200 pmol
Rev DNA template (Rev_dGsw[27])	1200 pmol
T7 RNA polymerase	30 µg/mL
YIPP	8 units
ddH_2O	Total volume 10 mL

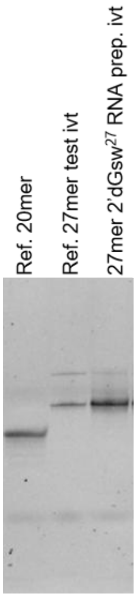

Figure S12 Denaturing polyacrylamide gel (20%) of the preparative in vitro transcription (prep. ivt) of the 27mer 2'dGsw[27] RNA. As References a test in vitro transcription of the 27mer 2'dGsw[27] RNA and a 20mer RNA were used. The run time was 55 min at 230 V. The gel was visualized via GelRed and Bio Rad ChemiDoc XRS+.

3.3.3 In vitro transcription optimization of the 2'dGsw[28-86] RNA

The DNA template and $Mg(OAc)_2$ optimization was performed as described in chapter 3.3.1. 6 pmol DNA template (in a 50 mL reaction volume, 1200 pmol in 10 mL) and 40 mM $Mg(OAc)_2$ were chosen for the preparative ivt.

The 2'dGsw[28-86] RNA requires a 5'-end mono phosphate for the following splinted ligation. For this purpose, guanosine monophosphate (GMP) was added to the ivt. GMP can only be incorporated at the

5'-end, where the transcriptions begins. Different GMP concentrations (1-10 eq.) were tested with the optimized DNA template and $Mg(OAc)_2$ conditions to evaluate the transcription efficiency with additional GMP. Therefore 50 µL test *in vitro* transcriptions as demonstrated in Table S15 were prepared. The transcriptions were incubated without the rNTPs, GMP and enzymes for 4 min and 80 °C and cooled within 10 min to 37 °C. Then the transcription mixture was incubated for 15 min at 37 °C and 300 rpm. Preheated rNTPs (37 °C, 15 min) and GMP (75°C, 15 min) were added. After that T7 RNA polymerase and YIPP were added and the mixture was incubated for 3 h at 37 °C. The results were analyzed by polyacrylamide gel electrophoreses (Figure S13). GMP did not affect the ivt efficiency. GMP equivalents 1 to 6 showed a similar good result. Whereas 8 and 10 had a by-product with low intensity over the desired product band. For the preparative ivt 3 eq. of GMP were chosen.

Table S15 In vitro transcription composition for the GMP optimization of the 59mer 2'dGsw$^{28\text{-}86}$ RNA.

	**Quantity**
Transcription buffer (200 mM tris/glutamate pH 8.1)	200 mM
DTT	20 mM
Spermidine	2 mM
$Mg(OAc)_2$	35 mM
DMSO	20%
rNTP mix	5 mM (1.25 mM each rNTP)
GMP	1.25, 2.5, 3.75,…,12.5 mM
Fwd DNA template (Fwd_dGsw$^{28\text{-}86}$)	2 pmol
Rev DNA template (Rev_dGsw$^{28\text{-}86}$)	2 pmol
T7 RNA polymerase	19.2 µg/mL
YIPP	0.01 Units
ddH_2O	Total volume 50 µL

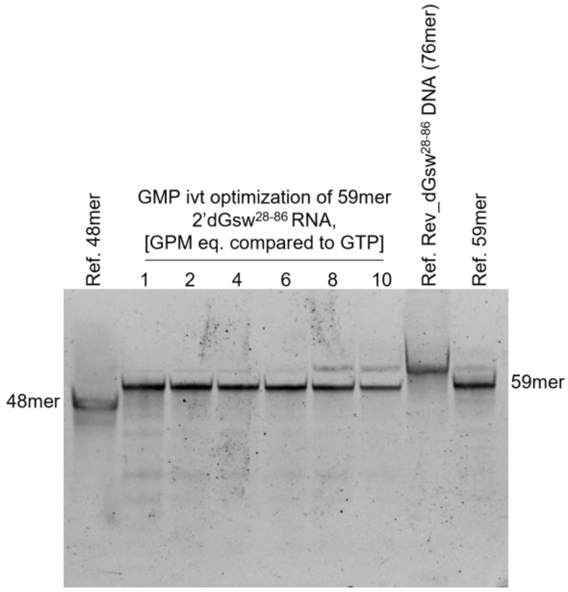

Figure S13 GMP ivt optimization of the 59mer 2'dGsw$^{28\text{-}86}$ RNA on a denaturing polyacrylamide gel (12%). Between 1 eq. and 10 eq. of GMP were used in the ivt compared to the GTP. As reference a 48mer & 59mer RNA and the 76mer Rev_dGsw$^{28\text{-}86}$ DNA template were applied on the gel. The run time was 45 min at 180 V. The gel was visualized via GelRed and Bio Rad ChemiDoc XRS+.

3.3.4 Preparative *in vitro* transcription of the 2’dGsw$^{28\text{-}86}$ RNA and purification

The preparative ivt of the 59mer 2’dGsw$^{28\text{-}86}$ RNA was prepared in a 10 mL reaction volume with optimized DNA template, $Mg(OAc)_2$ and GMP conditions according to Table S16. The DNA template annealing was performed as described in chapter 3.3.2. After T7 RNA polymerase addition the mixture was incubated at 37 °C and 70 rpm for 8 h. 1 h and 5 h after the start of the transcription 80 µL and 60 µL of YIPP were added. After the transcription buffer and remaining rNTP were removed with a centrifugal concentrator (Vivaspin™ 20 from *Sartorius*, 5000 MWCO). The RNA was analyzed by polyacrylamide gel electrophoreses (Figure S14). The yield of the 2’dGsw$^{28\text{-}86}$ RNA was 384.0 nmol.

Table S16 Composition for the preparative ivt of the 59mer 2’dGsw$^{28\text{-}86}$ RNA under optimized conditions. Nat. ab. means natural abundance.

	**Quantity**
Transcription buffer<br>(200 mM tris/glutamate pH 8.1)	200 mM
DTT	20 mM
Spermidine	2 mM
$Mg(OAc)_2$	40 mM
DMSO	20%
ATP nat. ab	3.6 mM
CTP nat. ab.	2.0 mM
GTP nat. ab.	2.4 mM
UTP nat. ab	2.0 mM
GMP nat. ab.	7.1 mM
Fwd DNA template (Fwd_dGsw[27])	1200 pmol
Rev DNA template (Rev_dGsw[27])	1200 pmol
T7 RNA polymerase	30 µg/mL
YIPP	8 units
ddH_2O	Total volume 10 mL

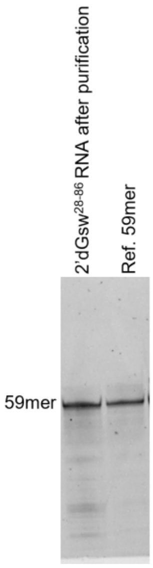

Figure S14 Denaturing polyacrylamide gel (15%) of the preparative in vitro transcription of the 59mer 2’dGsw$^{28\text{-}86}$ RNA after the purification. As references a 59mer test in vitro transcription of the 59mer 2’dGsw$^{28\text{-}86}$ RNA was applied on the gel. The run time was 45 min at 220 V. The gel was visualized via GelRed and Bio Rad ChemiDoc XRS+.

3.3.5 Optimization of the splinted ligation of the 2’dGsw^{27} and 2’dGsw$^{28-86}$ RNA

The 27mer 2’dGsw^{27} RNA was ligated with the 59mer 2’dGsw$^{28-86}$ RNA by the T4 RNA Ligase 2 in presence of a DNA splint. The 3’-end OH of the 2’dGsw^{27} RNA is connected with the 5’-end phosphate group of the 2’dGsw$^{28-86}$ RNA. Different ratios of both RNAs were tested to optimize the splinted ligation conditions. The tested conditions are shown in Table S17. The total reaction volume was 50 µL. The splinted ligation reaction mixture was incubated without enzyme at 80 °C for 4 min, then cooled to 37 °C within 10 min and further incubated at 37 °C and 300 rpm for 15 min. Thereafter T4 RNA Ligase 2 was added and the mixture incubated at 37 °C and 300 rpm for 3 h. Subsequently the DNase digestion was performed to remove the DNA splint. Therefore 5 µL 10x *Turbo™* DNase buffer and 1 µL (2 U) *Turbo™* DNase from *Invitrogen™* were added and the mixture was incubated at 37 °C for 90 min. The polyacrylamide gel electrophoreses with the results are demonstrated in Figure S15. The product gel band of the sample with an excess of 2’dGsw$^{28-86}$ (1/1.5/1 µM) has the strongest intensity compared to the other two samples. This ratio was used for the preparative splinted ligation.

Table S17 Splinted ligation optimization of the 27mer 2’dGsw^{27} and 59mer 2’dGsw$^{28-86}$ RNAs. An excess of both RNAs was tested. Reaction volume was 50 µL.

	**Quantity**
1) 2’dGsw^{27}/2’dGsw$^{28-86}$/Long DNA splint (84mer)	(1/1/1) µM
2) 2’dGsw^{27}/2’dGsw$^{28-86}$/Long DNA splint (84mer)	(1/1.5/1) µM
3) 2’dGsw^{27}/2’dGsw$^{28-86}$/Long DNA splint (84mer)	(1.5/1/1) µM
T4 DNA Ligase buffer	1x
In house produced T4 RNA Ligase 2	1% (v/v) added after annealing
ddH_2O	Filled up to 50 µL

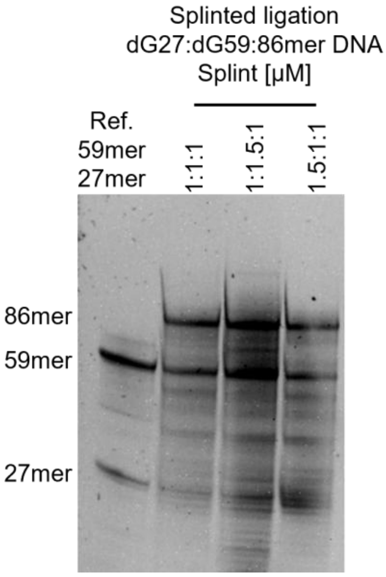

Figure S15 Splinted ligation optimization of the 2’dGsw^{27} and 2’dGsw$^{28-86}$ RNAs on a denaturing polyacrylamide gel (15%). Different ratios of both RNAs were tested. The run time was 44 min at 230 V. The gel was visualized via GelRed and Bio Rad ChemiDoc XRS+.

3.3.6 Preparative splinted ligation of the 2’dGsw^{27} and 2’dGsw$^{28\text{-}86}$ RNA to prepare the 2’dGsw^{86}-C26 RNA and NMR sample preparation

The preparative splinted ligation of the 2’dGsw^{27} and 2’dGsw$^{28\text{-}86}$ RNAs were performed under optimized conditions with an excess of 2’dGsw$^{28\text{-}86}$ RNA, see chapter 3.3.5. The pipetting scheme for the splinted ligation is demonstrated in Table S18. The DNA splint annealing, incubation and DNA splint digestion were performed as described in chapter 3.1.6. 120 reaction mixtures (each 250 µL) were prepared in total for the NMR sample. Increasing the volume reduces the ligation efficiency.[4] The RP-HPLC purification was performed at 60 °C with the column XBridge Peptide BEH C18 (300 Å, 3,5 µm, 4,6x250 mm) from Waters. RP-HPLC gradient is shown in Table S19. The RP-HPLC buffer was removed with a lyophilizer and the RNA pellets were reconstituted in ddH_2O. The fractions were analyzed by analytical denaturing PAGE and the fractions containing clean product were combined (Figure S16). The desired 2’dGsw^{86}-C26 RNA was predominantly obtained with a yield of 70 nmol (46%). 2 eq. (140 nmol) of ^{13}C,^{15}N-2’dG ligand and 40 eq. (2800 nmol) of $MgCl_2$ were added to the combined product fractions. Afterwards the RNA was folded by heating the sample to 80 °C for 30 sec and then placing it on ice for 10 min. Then the NMR sample was dried in a vacuum concentrator and reconstituted in 15 µL NMR-Buffer (50 mM KCl, 25 mM K_2HPO_4/KH_2PO_4 at pH 6.2). 15 µL d8-glycerol and 0.3 µmol AsymPolPOK were added to yield a final total concentration of 10 mM AsymPolPOK. The solution was transferred to a 3.2 mm Sapphire MAS NMR rotor and a small Teflon insert was added before closing the rotor.

Table S18 Pipetting scheme for a single splinted ligation mixture of the 2’dGsw^{27} and 2’dGsw$^{28\text{-}86}$ RNAs. For the NMR sample 120 reactions mixtures were splint ligated.

	**Quantity**
2’dGsw^{27} RNA (27mer)	5 µM
2’dGsw$^{28\text{-}86}$ RNA (59mer)	6 µM
Long DNA splint (84mer)	5 µM
T4 DNA Ligase buffer	1x
In house produced T4 RNA Ligase 2	1% (v/v) added after annealing
ddH_2O	Filled up to 250 µL

Table S19 RP-HPLC Gradient for the purification of the 2’dGsw^{86}-C26 RNA after splinted ligation and DNA splint digestion. Hexafluoro-2-propanol (HFIP) buffer was prepared with 0.4 M HFIP and 16.3 mM triethylamine (TEA) in ddH_2O. XBridge Peptide BEH C18 (300 Å, 3,5 µm, 4,6x250 mm) column from Waters was used. The RP-HPLC was performed at 60 °C.

Time	0.4 M HFIP buffer (%)	Methanol (%)
0	95	5
3	95	5
8	82	18
35	71	29
38	0	100

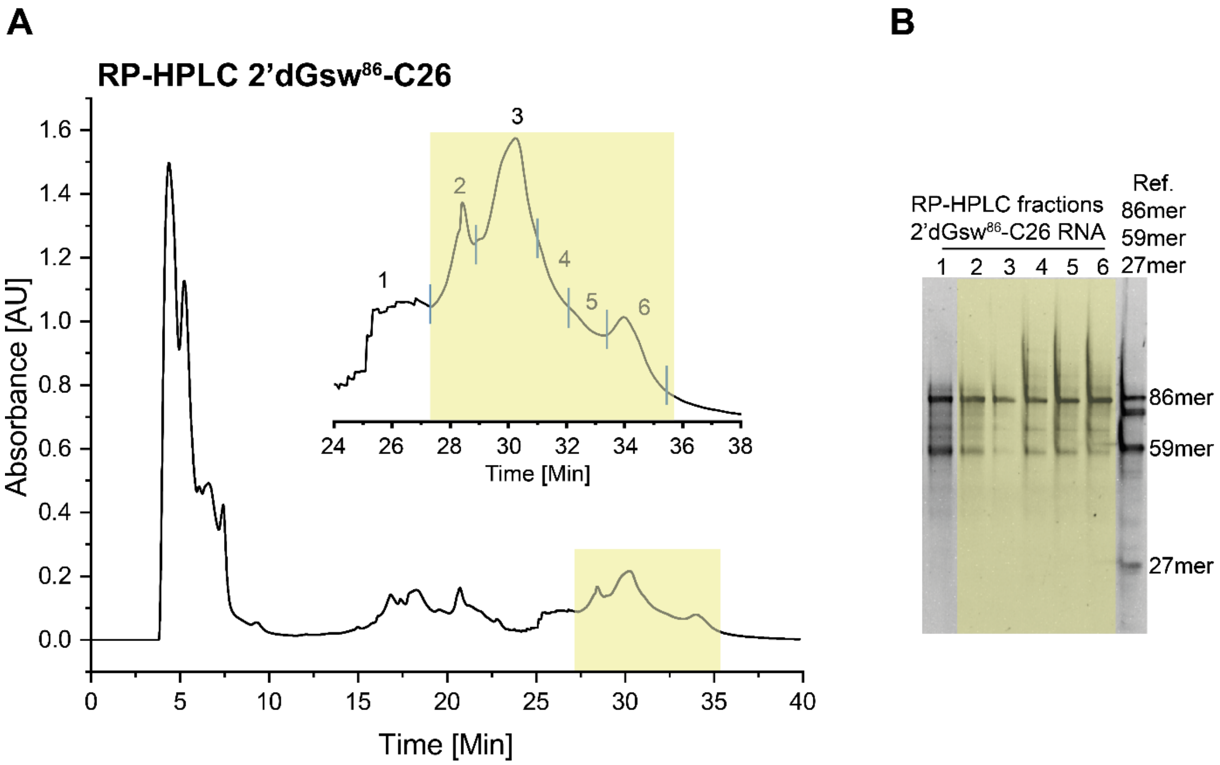

Figure S16 ***A****) RP-HPLC chromatogram of the 2'dGsw*^{86}*-C26 RNA purification. The product RNA signals are highlighted in yellow and have a retention time of ca. 28.0-35.0 min.* ***B****) Denaturing polyacrylamide gel (15%) of the RP-HPLC purification of the 2'dGsw*^{86}*-C26 RNA. The band below the 86mer reference is a degradation by-product of the 86mer. The run time was 45 min at 230 V.*

3.4 Synthesis and quality control of the 2'dGsw^{70}-A25 RNA

The 2'dGsw^{70}-A25 RNA was synthesized by *Innotope*. The Sequence is shown in Table S4. The chemical RNA synthesis is described in chapter 3.4.1. The quality control of the delivered product was performed by polyacrylamide gel electrophoreses, see chapter 3.4.2.

3.4.1 Chemical RNA Synthesis of the 2'dGsw^{70}-A25 RNA and Mass Spectrometry

Sequence of the 2'dG^{70}-A25 (70mer) was synthesized on an ABI 391 PCR Mate using standard 2'-O-TBDMS RNA phos-phoramidites (rA^{Ac}, rG^{Ac}, rC^{Ac}, rU - *ChemGenes*) together with 1',2,8-$^{13}C_3$-rA^{Ac}-2'-O-TBDMS phosphoramidite (*Innotope GmbH*) and rC^{Ac}-CPG solid support (27 µmol g^{-1}, 1000 Å, *GlenResearch*) employing an RNA synthesis cycle (approx. 6 min coupling time).

Amidites (100 mM in acetonitrile, ACN) and activator (BTT, 5-benzylthio-1H-tetrazole, 250 mM in ACN) were dried for 48 hours over activated 3 Å molecular sieves.

Reagent mixtures used:

- Detritylation solution: 4% dichloroacetic acid in toluene.
- Capping solutions:
    - Cap A: 800 mM 2,6-lutidine, 1.0 M ace-tic anhydride in THF
    - Cap B: 200 mM N-methylimidazole in THF.
- Oxidation solution: 0.02 M I_2 in THF/pyridine/H2O (7:2:1, v/v/v)

After the RNA synthesis, the solid support was dried under high vacuum for 10 minutes, then pretreated with 100 mL 20% diethylamine in ACN for 30 minutes, rinsed with 80 mL ACN, and dried again under high vacuum for 30 minutes.

The solid support was then incubated under AMA-conditions (1 mL aq. NH_3, 28-30%, *Merck*; and 1 mL aq. $MeNH_2$, 40%, *Merck*) for 5 hours at 37 °C. The solid support was filtered off, and the filtrate was collected in a 10 mL round-bottom flask. The support washed three times with THF/H_2O (1:1, v/v) and the combined filtrates were evaporated by means of a rotary evaporator at 45 °C. The dry residue was further dried under high vacuum for 1 hour.

The residue was dissolved in 300 µL anhydrous DMSO, followed by the addition of 375 µL TEA·3HF (*Merck*), and incubated at 37 °C for 16 hours. The deprotection was quenched with 3 mL quenching buffer (*GlenResearch*) and directly desalted via size exclusion chromatography (SEC) on a *Äkta™Start* (*GE-Healthcare*) equipped with a HiPrep™ 26/10 Desalting Column (*Cytiva*).

The crude RNA was purified via anion-exchange chromatography on a Dionex DNAPac PA-200 column (22x250 mm, *ThermoScientific*) at 80 °C.

- Buffer A: 25 mM Tris-HCl pH 8.0, 10 mM $NaClO_4$, 20% ACN (v/v).
- Buffer B: 25 mM Tris-HCl pH 8.0, 600 mM $NaClO_4$, 20% ACN (v/v).

The pooled fractions were concentrated by means of a rotary evaporator at 45 °C to remove the ACN-content, diluted 1:1 (v/v) with 100 mM NaCl buffer and loaded on a C18 Seq-Pak cartridge (*Waters*) to remove HPLC buffer salts. The pure RNA (sodium salt form) was eluted with water/ACN (1:1, v/v) and lyophilized.

The synthesized RNA was analyzed on *Finnigan LCQ Advantage MAX* ion trap instrumentation connected to a *Thermo Scientific UHPLC* (components: *Ultimate 3000 RS* Pump, *Ultimate 3000 RS* Autosampler, *Ultimate 3000 RS* Column Compartment, *Ultimate 3000* Diode Array Detector). RNA mass spectra were acquired in the negative-ion mode with a potential -4kV applied to the spray needle (capillary voltage: -23V, capillary temperature: 270 °C), see Figure S17. LC: 1 µL of dissolved RNA in 29 µL of 20 mM ethylenediaminetetraacetic acid (EDTA) solution; average injection volume: 30 µL; column: *Waters xBridge* C18 2.5 µm column (1.0 x 50 mm) at 30 °C; flow rate: 100 µL/min; Eluent A: 8.6 mM triethylamine (TEA), 100 mM 1,1,1,3,3,3-hexafluoroisopropanol in H_2O (pH 8.0); Eluent B: methanol; gradient: 0-100% B in A within 30 min; UV detection was carried out at 260/280nm.

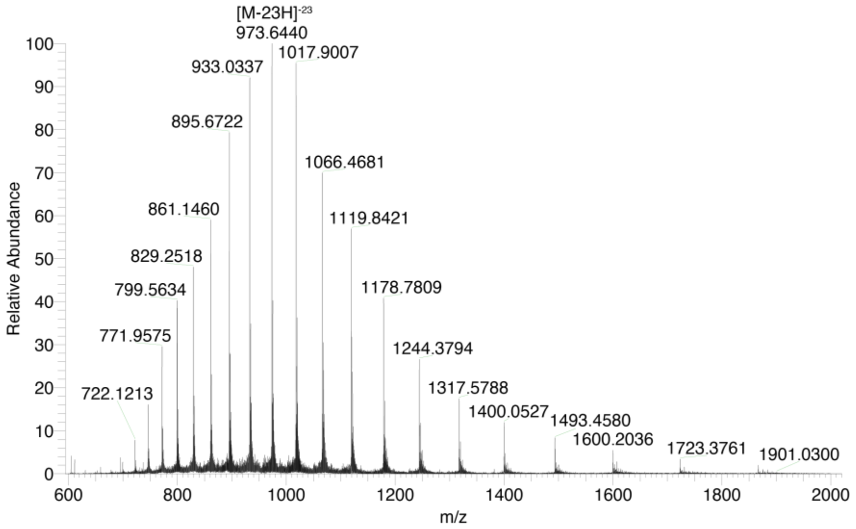

Figure S17 Mass spectrum of the 2’dGsw^{70}-A25 RNA after purification. Calculated mass: 22.41 kDa., found 973.6440 $[M-23H]^{-23}$.

3.4.2 Quality control and NMR sample preparation

RNA purity of the delivered product was analyzed by polyacrylamide gel electrophoreses (Figure S18). The RNA was pure, no impurities or by-products were observed.

For the NMR sample preparation 110 nmol 2’dGsw^{70}-A25 RNA were mixed with 2 eq. (220 nmol) of ^{15}N-2’dG ligand and 40 eq. (4400 nmol) of $MgCl_2$. Then the RNA was folded by heating the sample to 80 °C for 30 sec and then placing it on ice for 10 min. Afterwards the NMR sample was dried in a vacuum concentrator and reconstituted in 15 µL NMR-Buffer (50 mM KCl, 25 mM K_2HPO_4/KH_2PO_4 at pH 6.2). 15 µL d8-glycerol and 0.3 µmol AsymPolPOK were added to yield a final total concentration of 10 mM AsymPolPOK. The solution was transferred to a 3.2 mm Sapphire MAS NMR rotor and a small Teflon insert was added before closing the rotor.

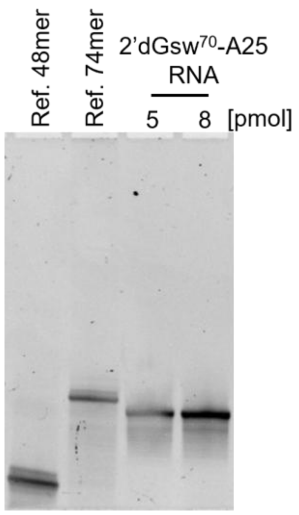

Figure S18 Denaturing polyacrylamide gel (10%) of the purchased 2’dGsw^{70}-A25 RNA from Innotope for quality control. The RNA shows no impurities or by-products. As reference a 74mer and 48mer RNA was used. The run time was 35 min at 240 V. The gel was visualized via GelRed and Bio Rad ChemiDoc XRS+.

3.5 Preparation of the 2’dGsw^{86}-C75-(5-D, 1’,6-^{13}C) RNA with a splinted ligation

The 2’dGsw^{86}-C75-(5-D, 1’,6-^{13}C) RNA was synthesized with a splinted ligation of two RNA fragments the 2’dGsw^{74} and 2’dGsw$^{75-86}$ RNA (Figure S19). The 2’dGsw^{74} RNA was prepared by *in vitro* transcription (ivt) and the 2’dGsw$^{75-86}$ RNA with a 5-D, 1’,6-^{13}C-cytidine modification was synthesized by *Innotope*. The sequences of the RNA fragments, DNA splint and DNA primers are demonstrated in Table S5.

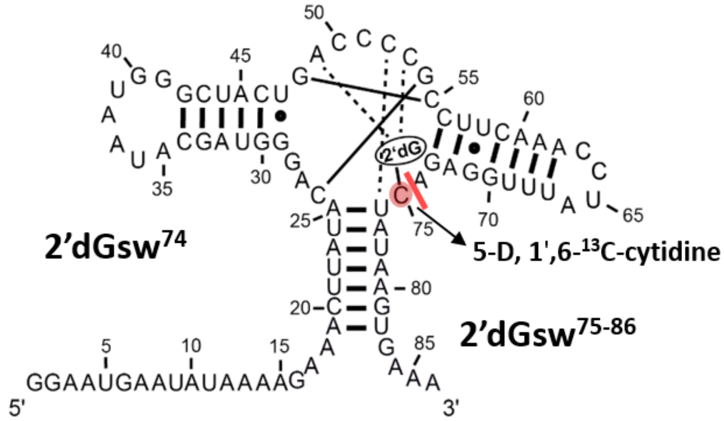

Figure S19 Secondary structure of the 2’dGsw^{86}-C75-(5-D, 1’,6-^{13}C) RNA with a 5-D, 1’,6-^{13}C-cytidine in position 75. The red line demonstrates where the two RNA fragments (2’dGsw^{74} and 2’dGsw$^{75-86}$) were splint ligated.

3.5.1 Preparation of the 2’dGsw^{74} RNA fragment for splinted ligation

The 2’dGsw^{74} RNA preparation was performed as described in chapters 3.1.1 and 3.1.2. The ivt was done twice. The combined yield after purification with centrifugal concentrator was 451 nmol.

3.5.2 Quality control of the 2’dGsw$^{75-86}$ RNA

The 2’dGsw$^{75-86}$ RNA with a 5-D, 1’,6-^{13}C-cytidine at the 5’-end was synthesized by *Innotope*. Before splinted ligation the RNA was analyzed by polyacrylamide gel electrophoreses (Figure S20). Only the product band could be observed on the gel image. No impurities or by-products were detected. The intensity of the product band is weak, because the dye does not intercalate well due to the short length of 12 nt. The amount of the 2’dGsw$^{75-86}$ RNA was 444 nmol.

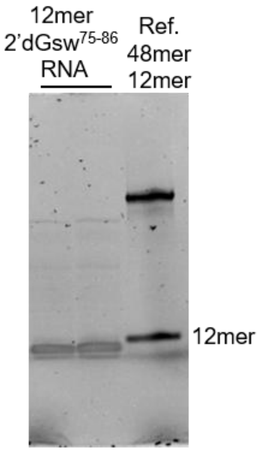

Figure S20 Quality control of the 2'dGsw$^{75\text{-}86}$ RNA synthesized by Innotope on a denaturing polyacrylamide gel (20%). The 2'dGsw$^{75\text{-}86}$ RNA band has a weak intensity, because it seems like the dye does not intercalate well due to the short length of 12 nt. The 12mer 2'dGsw$^{75\text{-}86}$ RNA migrates slightly faster than the 12mer reference RNA. This is caused by the different sequence of the nucleotides, which plays a role in the migration speed in the gel for short RNAs. The run time was 40 min at 220 V. The gel was visualized via GelRed and Bio Rad ChemiDoc XRS+.

3.5.3 Preparative splinted ligation of the 2'dGsw^{74} and 2'dGsw$^{75\text{-}86}$ RNA to prepare the 2'dGsw^{86}-C75-(5-D, 1',6-^{13}C) RNA

The preparative splinted ligation of the 2'dGsw^{74} and 2'dGsw$^{75\text{-}86}$ RNAs were performed with an excess of the 2'dGsw$^{75\text{-}86}$ RNA. The pipetting scheme for the splinted ligation is demonstrated in Table S20. The DNA splint annealing, incubation and DNA splint digestion were performed as described in chapter 3.1.6. 188 reaction mixtures (each 250 µL) were prepared in total for the NMR sample. Increasing the volume reduces the ligation efficiency.[4] The splinted ligation efficiency was high. Both RNAs fragments were mostly converted to the desired 2'dGsw^{86}-C75-(5-D, 1',6-^{13}C) RNA. An aliquot of the product was purified by RP-HPLC (Figure S21 **C** and **D**). However, the separation of the 74mer 2'dGsw^{74} RNA fragment and the 86mer 2'dGsw^{86}-C75-(5-D, 1',6-^{13}C) RNA product was not successful. Hence the product was purified by centrifugal concentrator (Vivaspin^{TM} 20 from *Sartorius*, 5000 MWCO). The residual 12mer 2'dGsw$^{75\text{-}86}$ RNA will pass the membrane and the 74mer 2'dGsw^{74} RNA fragment has no labelled atoms and is present in small quantites and therefore will not interfere with the product. In Figure S21 **B** the product 2'dGsw^{86}-C75-(5-D, 1',6-^{13}C) RNA is shown after purification. The desired product was predominantly obtained with a yield of 200 nmol (65%). For the NMR sample preparation, the RNA amount was reduced to 120 nmol due to solubility problems in a volume of 15 µL ddH_2O. 2 eq. (240 nmol) of ^{15}N-2'dG ligand and 40 eq. (4800 nmol) of $MgCl_2$ were added. Then the RNA was folded by heating the sample to 80 °C for 30 sec and then placing it on ice for 10 min. Afterwards the NMR sample was dried in a vacuum concentrator and reconstituted in 15 µL NMR-Buffer (50 mM KCl, 25 mM K_2HPO_4/KH_2PO_4 at pH 6.2). 15 µL d8-glycerol and 0.3 µmol AsymPolPOK were added to yield a final total concentration of 10 mM AsymPolPOK. The solution was transferred to a 3.2 mm Sapphire MAS NMR rotor and a small Teflon insert was added before closing the rotor.

Table S20 Pipetting scheme for a single splinted ligation mixture of the 2’dGsw^{74} and 2’dGsw$^{75\text{-}86}$ RNAs. For the NMR sample 188 reactions mixtures were splint ligated.

	**Quantity**
2’dGsw^{74} RNA (74mer)	6.5 µM
2’dGsw$^{75\text{-}86}$ RNA (12mer)	8.45 µM
Short DNA splint (42mer)	6.5 µM
T4 DNA Ligase buffer	1x
In house produced T4 RNA Ligase 2	1.3% (v/v) added after annealing
ddH$_2$O	Filled up to 250 µL

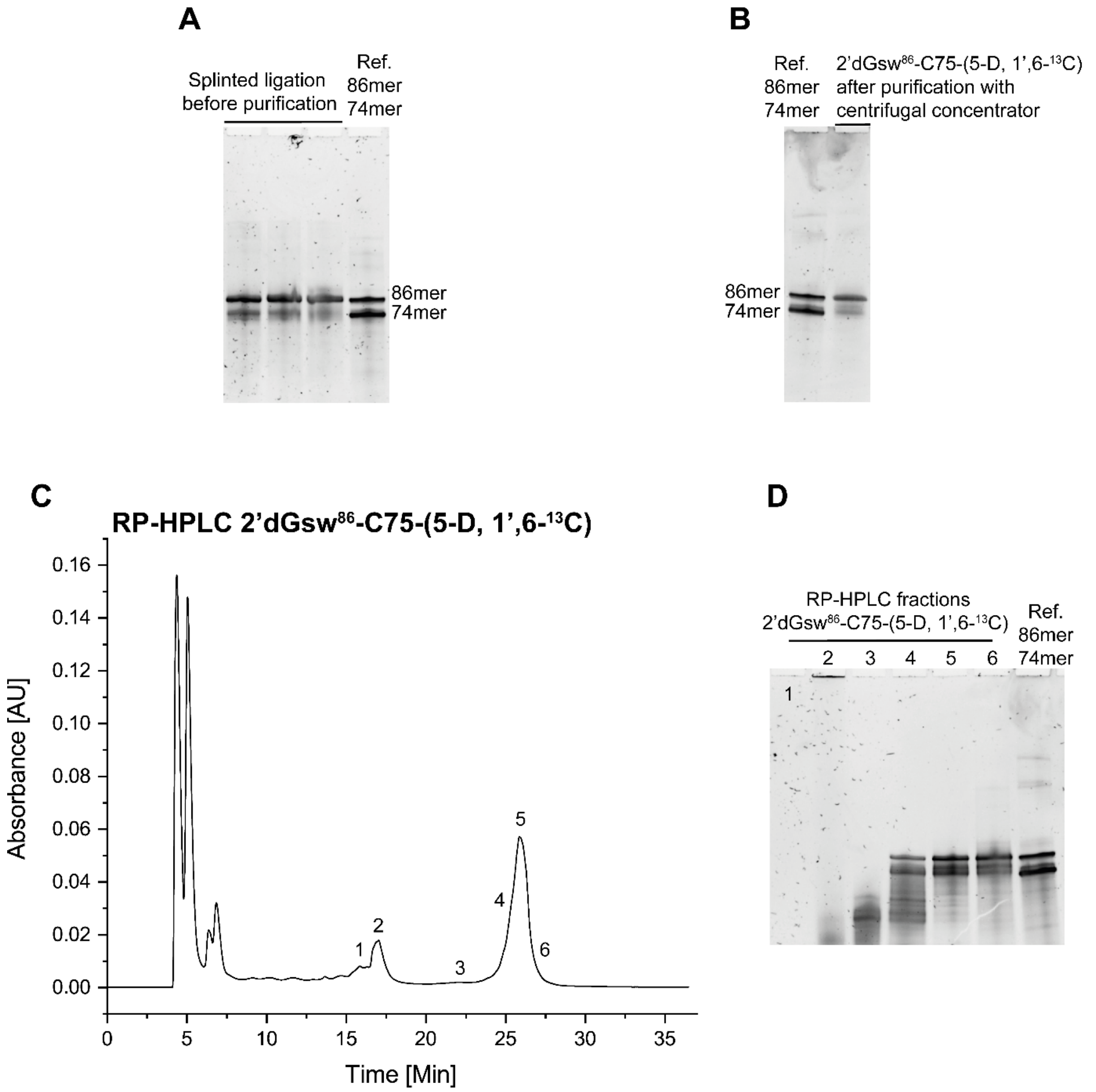

Figure S21 ***A)*** *Splinted ligation product of 2’dGsw^{74} and 2’dGsw$^{75\text{-}86}$ RNAs before purification on a denaturing polyacrylamide gel (8%). The starting material was almost completely converted to the desired product 2’dGsw^{86}-C75-(5-D, 1’,6-^{13}C) RNA.* ***B)*** *Denaturing polyacrylamide gel (8%) of 2’dGsw^{86}-C75-(5-D, 1’,6-^{13}C) RNA after purification by centrifugal concentrator.* ***C)*** *RP-HPLC chromatogram of an aliquot of the 2’dGsw^{86}-C75-(5-D, 1’,6-^{13}C) splinted ligation mixture.* ***D)*** *Denaturing polyacrylamide gel (8%) of the RP-HPLC fractions. Fractions 4, 5 and 6 contain the product. However, the product RNA could not be separated from the 74mer 2’dGsw^{74} RNA. The run time for all gels was 30 min at 240 V.*

4. Solid state MAS-DNP measurements

4.1 MAS DNP NMR experiments

Most measurements were performed with a 400 MHz/263 GHz solid-state DNP Avance Neo spectrometer from Bruker equipped with a 3.2mm Cryo MAS HCN probe head. Spectra on the ^{15}N-2'dG and 2'dGsw^{86}-C75-(5-D, 1',6-^{13}C) sample were recorded on a similar spectrometer (using a Avance III spectrometer consol instead of Avance NEO) at the TU Darmstadt. All spectra were recorded at a temperature of around 110 K and at a MAS frequency of 10417 Hz. A recycle delay of 1 s was used except for the TEDOR spectra with a mixing time longer than 20 ms for which a recycle delay of 1.5 s was used to take the duty cycle of the probe into account. ^{1}H decoupling during detection and the TEDOR mixing step was 100 kHz. An overview of the experiment parameters can be found in Table S21.

For the [^{15}N-2'dG and 2'dGsw^{70}-A25] NMR sample the ^{13}C-T_2 times of the A25 were determined using a Hahn Echo experiment, see Figure S22.

Table S21 Overview of the TEDOR experiment parameters. The 2'dGsw^{86}-C26/C53/C75 RNA samples have a single site-specific ^{13}C,^{15}N labeled cytidine. The A25 has ^{13}C labeled atoms at position 1',2 and 8.

Sample	DNP-enhancement	^{1}H-T_1	TEDOR t_{mix}	TEDOR d1	TEDOR ns	TEDOR number of indirect data points (indirect evolution time	TEDOR experimental time
^{13}C,^{15}N-2'dG	100	0.4 s	1.142 ms	1 s	128	100 (4.8 ms)	3 h, 39 min
^{13}C,^{15}N-2'dG and 2'dGsw^{86}	94	0.5 s	1.142 ms	1 s	896	64 (3.0 ms)	16 h, 20 min
^{13}C,^{15}N-2'dG and 2'dGsw^{86}-C26	64	0.5 s	1.142 ms	1 s	1024	64 (3.0 ms)	18 h, 39 min
^{13}C,^{15}N-2'dG and 2'dGsw^{86}-C53	68	0.6 s	1.142 ms	1 s	1024	64 (3.0 ms)	18 h, 39 min
^{13}C,^{15}N-2'dG and 2'dGsw^{86}-C75	66	0.7 s	1.142 ms	1 s	1152	64 (3.0 ms)	20 h, 59 min
^{15}N-2'dG and 2'dGsw^{70}-A25	70	0.7 s	2.688 ms	1 s	2048	80 (3.8 ms)	46 h, 49 min
			4.992 ms	1 s	2048	80 (3.8 ms)	46 h, 56 min
			8.832 ms	1 s	2048	80 (3.8 ms)	47 h, 6 min
			12.672 ms	1 s	2048	80 (3.8 ms)	47 h, 16 min
			16.512 ms	1 s	2048	80 (3.8 ms)	47 h, 27 min
			20.352 ms	1.5 s	2048	80 (3.8 ms)	70 h, 23 min
			24.192 ms	1.5 s	2048	80 (3.8 ms)	70 h, 34 min
			28.032 ms	1.5 s	2048	80 (3.8 ms)	70 h, 44 min
^{15}N-2'dG and 2'dGsw^{86}-C75-(5-D, 1',6-^{13}C)	76	0.6 s	11,904 ms	1 s	2560	40 (1.9 ms)	35 h, 20 min

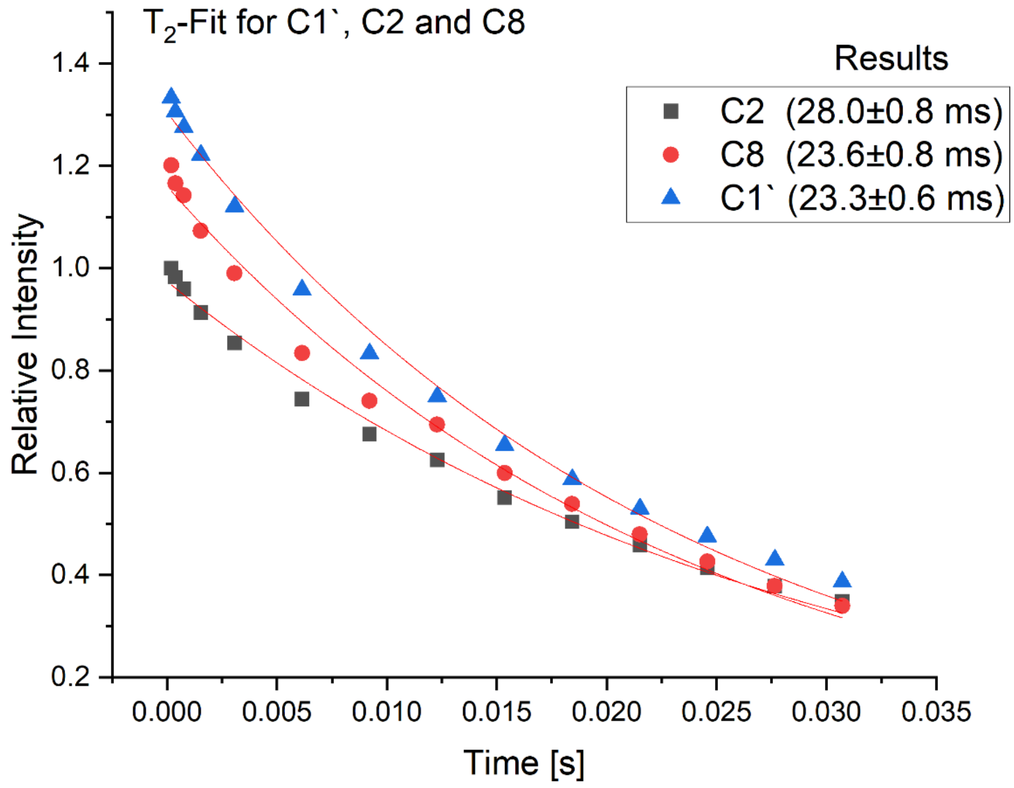

Figure S22 ^{13}C-T_2 time determination of the C1`, C2 and C8 atoms of the A25 in the [^{15}N-2'dG and 2'dGsw^{70}-A25] NMR sample. A Hahn Echo experiment was used for the ^{13}C-T_2 times determination.

4.2 MAS-DNP TEDOR-analysis

To obtain the integrals for the TEDOR built-up curves on the 2'dGsw^{70}-A25 RNA sample, the 1D projections of the labeled ^{13}C atoms were calculated and the obtained 1Ds were integrated at the position of the 5 ^{15}N atoms. The obtained intensities were then corrected for the differences in d1 and for differences in the microwave intensities as obtained from 1D TEDOR at t_{mix}=16.512 ms spectra that were recorded before the respective 2D experiments.

For each ^{13}C chemical shift all built-up curves were fit simultaneously using the equations described in Jaroniec et al.[7] using Wolfram Mathematica (version 13.3). The experimentally determined T_2 times for each of the three carbon atoms were used when fitting the data (Figure S22). Due to the very small signal intensities observed for the cross peaks with N2 only the intensities of 2'dG nitrogens N1, N3, N7 and N9 were used in the global fit. The following code (Figure S23) was used for the fitting procedure as shown on the example of the cross peaks at the chemical shift of ^{13}C2-2'dGsw^{70}-A25. From the obtained dipolar couplings the interatomic distances were calculated and reported errors correspond to the 95% confidence interval of the fitting. The range of acceptable fits in a 95% confidence interval is demonstrated in Figure S24.

The obtained 95% confidence intervals were then used as the lower and upper distance limits for the structure calculation in CYANA 3.98.15 software.[8] The sequence of two residues (Adenosine and Guanosine), connected by a flexible linker of dummy atoms was created to run the calculations.

2’dGsw^{70}-A25 - ^{13}C2

```
DistC2N9 = {{0, 0}, {28, 0.29724}, {52, 0.78129}, {92, 1.51053}, {132, 1.76629}, {172, 1.68179}, {212, 1.28818}, {252, 0.88618}, {292, 0.62668}}

DistC2N7 = {{0, 0}, {28, 0.22537}, {52, 0.56416}, {92, 1.12742}, {132, 1.23106}, {172, 1.19497}, {212, 0.8415}, {252, 0.64311}, {292, 0.4367}}

DistC2N1 = {{0, 0}, {28, 0.07793}, {52, 0.24822}, {92, 0.47764}, {132, 0.53906}, {172, 0.47119}, {212, 0.41346}, {252, 0.28114}, {292, 0.21289}}

DistC2N3 = {{0, 0}, {28, 0.11963}, {52, 0.27595}, {92, 0.54906}, {132, 0.67444}, {172, 0.60429}, {212, 0.51937}, {252, 0.39334}, {292, 0.32629}}

DAMPN9[DN9_, DN7_, DN1_, DN3_, mas_, m_, n_, k_, lb_, A_] :=
  A * Exp[-n * N[Pi] * (1 / mas) * lb] * (1 - N[BesselJ[0, N[Sqrt[2.0] (m + n) * 0.5 * DN9 / mas]]]^2) * (1 + N[BesselJ[0, N[Sqrt[2.0] (m + n) * 0.5 * DN7 / mas]]]^2) *
   (1 + N[BesselJ[0, N[Sqrt[2.0] (m + n) * 0.5 * DN1 / mas]]]^2) * (1 + N[BesselJ[0, N[Sqrt[2.0] (m + n) * 0.5 * DN3 / mas]]]^2)

DAMPN7[DN9_, DN7_, DN1_, DN3_, mas_, m_, n_, k_, lb_, A_] :=
  A * Exp[-n * N[Pi] * (1 / mas) * lb] * (1 - N[BesselJ[0, N[Sqrt[2.0] (m + n) * 0.5 * DN7 / mas]]]^2) * (1 + N[BesselJ[0, N[Sqrt[2.0] (m + n) * 0.5 * DN9 / mas]]]^2) *
   (1 + N[BesselJ[0, N[Sqrt[2.0] (m + n) * 0.5 * DN1 / mas]]]^2) * (1 + N[BesselJ[0, N[Sqrt[2.0] (m + n) * 0.5 * DN3 / mas]]]^2)

DAMPN1[DN9_, DN7_, DN1_, DN3_, mas_, m_, n_, k_, lb_, A_] :=
  A * Exp[-n * N[Pi] * (1 / mas) * lb] * (1 - N[BesselJ[0, N[Sqrt[2.0] (m + n) * 0.5 * DN1 / mas]]]^2) * (1 + N[BesselJ[0, N[Sqrt[2.0] (m + n) * 0.5 * DN9 / mas]]]^2) *
   (1 + N[BesselJ[0, N[Sqrt[2.0] (m + n) * 0.5 * DN7 / mas]]]^2) * (1 + N[BesselJ[0, N[Sqrt[2.0] (m + n) * 0.5 * DN3 / mas]]]^2)

DAMPN3[DN9_, DN7_, DN1_, DN3_, mas_, m_, n_, k_, lb_, A_] :=
  A * Exp[-n * N[Pi] * (1 / mas) * lb] * (1 - N[BesselJ[0, N[Sqrt[2.0] (m + n) * 0.5 * DN3 / mas]]]^2) * (1 + N[BesselJ[0, N[Sqrt[2.0] (m + n) * 0.5 * DN9 / mas]]]^2) *
   (1 + N[BesselJ[0, N[Sqrt[2.0] (m + n) * 0.5 * DN7 / mas]]]^2) * (1 + N[BesselJ[0, N[Sqrt[2.0] (m + n) * 0.5 * DN1 / mas]]]^2)

DataForFit =
  Flatten[Table[{{DistC2N9⟦i⟧⟦1⟧, 1, DistC2N9⟦i⟧⟦2⟧}, {DistC2N7⟦i⟧⟦1⟧, 2, DistC2N7⟦i⟧⟦2⟧}, {DistC2N1⟦i⟧⟦1⟧, 3, DistC2N1⟦i⟧⟦2⟧},
     {DistC2N3⟦i⟧⟦1⟧, 4, DistC2N3⟦i⟧⟦2⟧}}, {i, Length[DistC2N9]}], 1]

mas = 10417;
k = 5;
lb = 11.37;

ModelForFit[DN9_, DN7_, DN1_, DN3_, A_, m_, Num_] :=
  Piecewise[{{DAMPN9[DN9, DN7, DN1, DN3, mas, m, m, k, lb, A], Num == 1}, {DAMPN7[DN9, DN7, DN1, DN3, mas, m, m, k, lb, A], Num == 2},
    {DAMPN1[DN9, DN7, DN1, DN3, mas, m, m, k, lb, A], Num == 3}, {DAMPN3[DN9, DN7, DN1, DN3, mas, m, m, k, lb, A], Num == 4}}]

nlm = NonlinearModelFit[DataForFit, ModelForFit[DN9, DN7, DN1, DN3, A, x, Num], {{DN9, 75}, {DN7, 50}, {DN1, 50}, {DN3, 50}, {A, 50}}, {x, Num}]

FittedModel[ {«1»} ]

nlm["ParameterTable"]
```

Figure S23 Wolfram Mathematica code for the distance calculation between the ^{13}C2 of the A25 of the 2’dGsw^{70}-A25 and the ^{15}N at position N1, N3, N7 and N9 of the ligand.

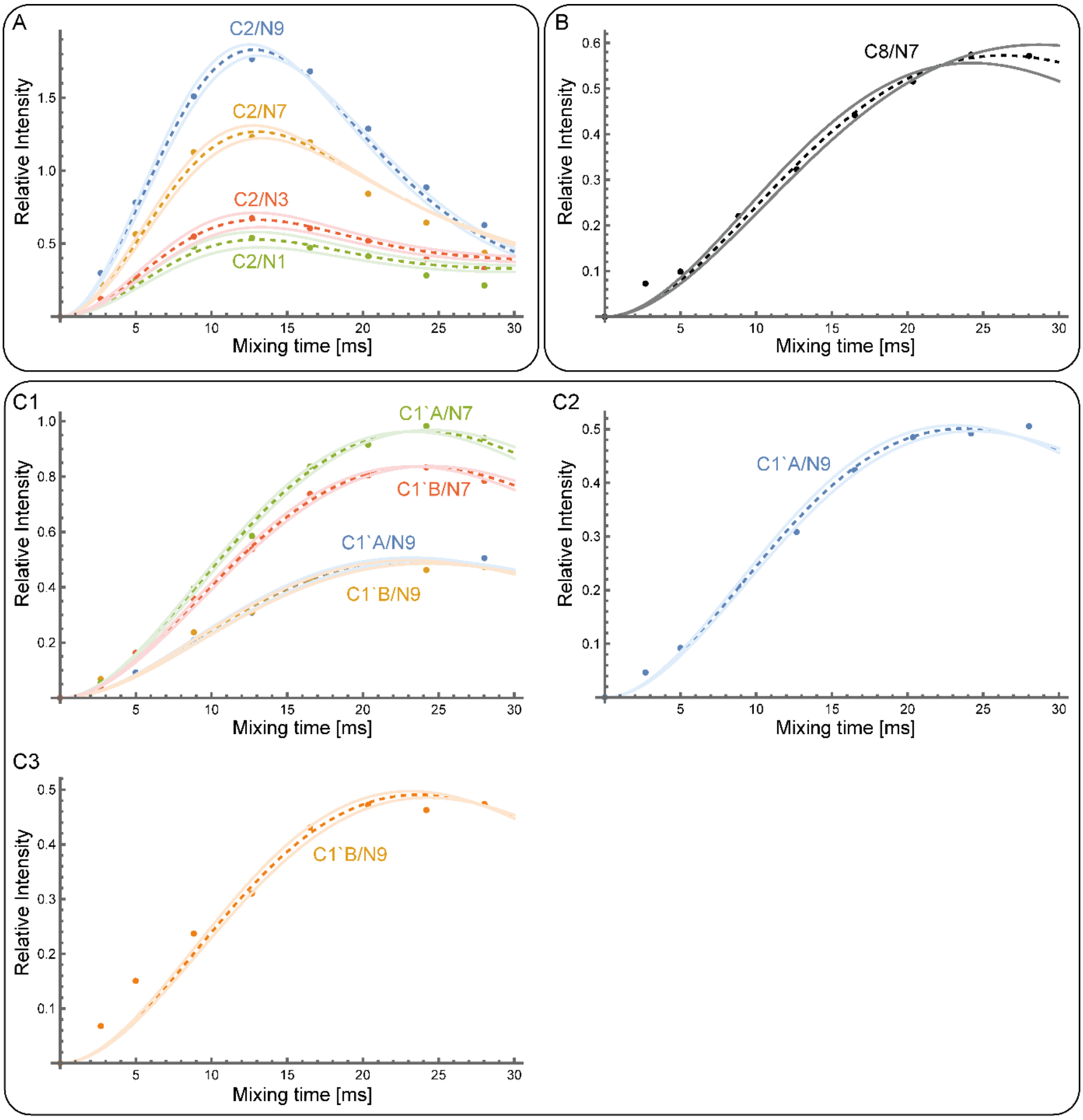

*Figure S24 The TEDOR cross-peak intensities measured for the **A**) C2, **B**) C8 and **C1-3**) C1' of the adenosine (^{13}C) and the N1, N3, N7 and N9 of the ligand (^{15}N) for the 2'dGsw^{70}-A25 Sample. Approximation of the experimental data by theoretical dependence is shown by dashed lines. The range of acceptable fits in a 95% confidence interval is shown by solid lines. **C2-3**) show the C1'A/N9 and C1'A/N9 fits separately, as they overlap in **C1**).*

4.3 NMR spectra

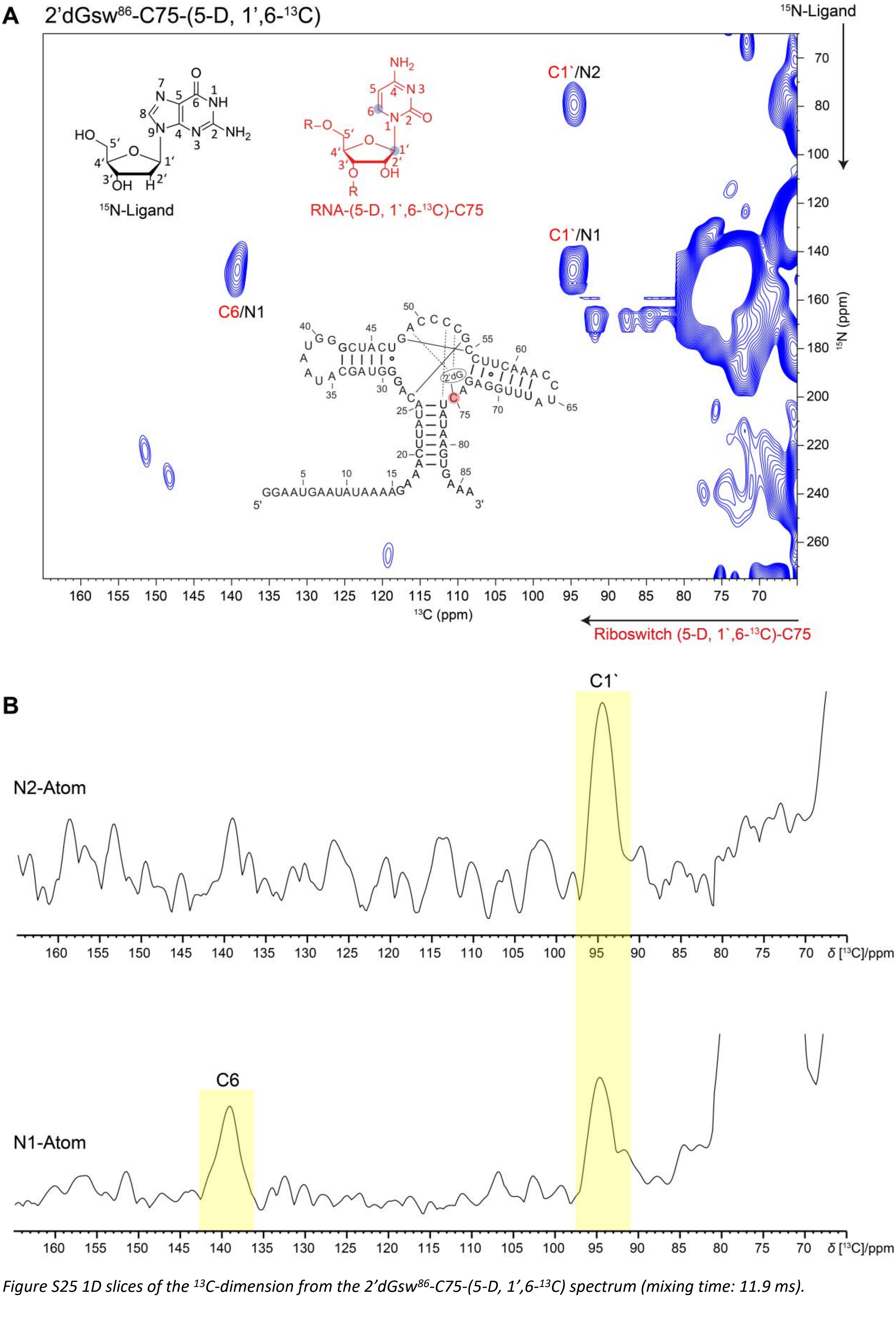

Figure S25 1D slices of the ^{13}C-dimension from the 2'dGsw^{86}-C75-(5-D, 1',6-^{13}C) spectrum (mixing time: 11.9 ms).

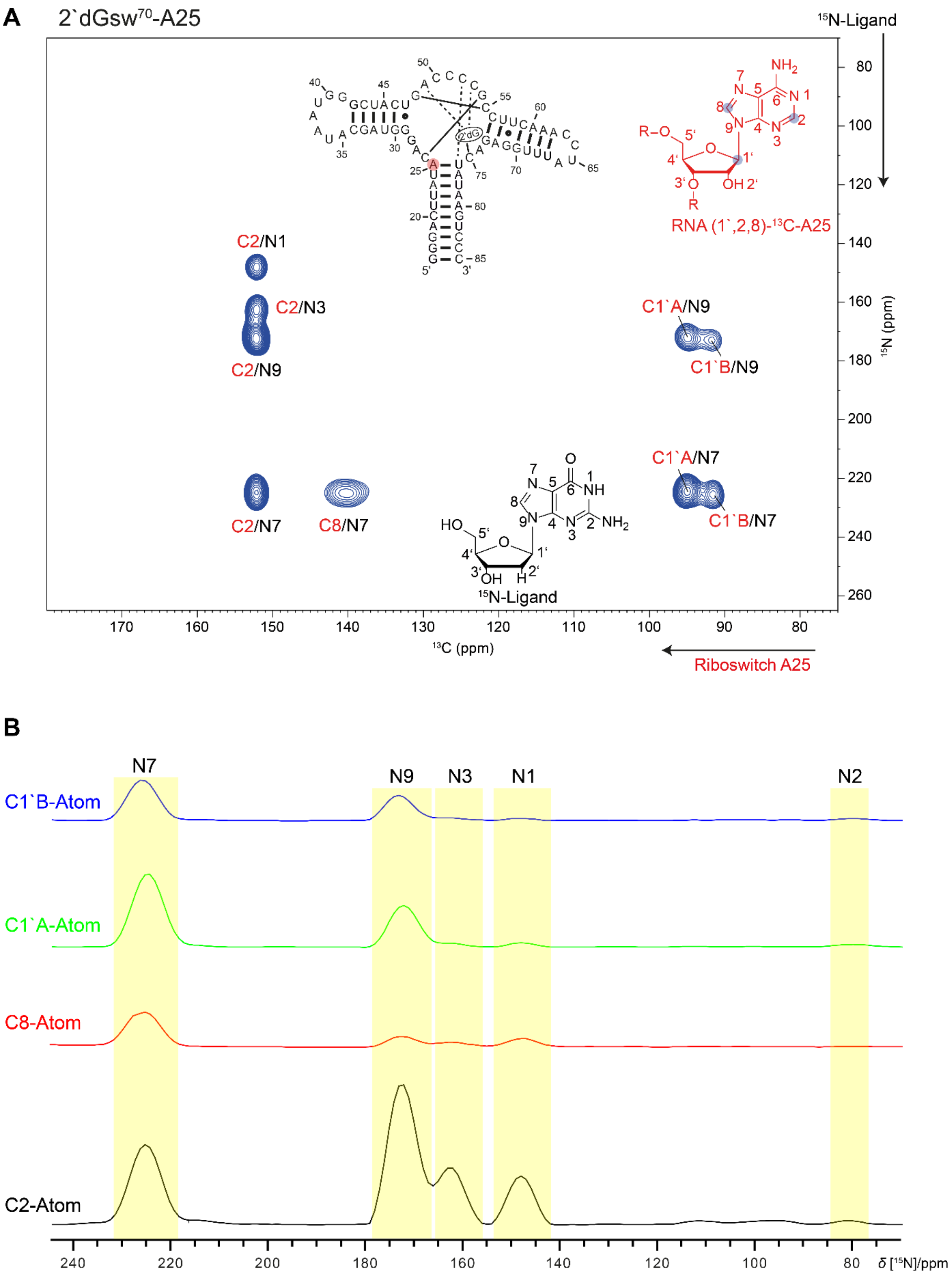

Figure S26 1D slices of the ^{15}N-dimension from the 2’dGsw^{70}-A25 RNA spectrum (mixing time: 20.4 ms).

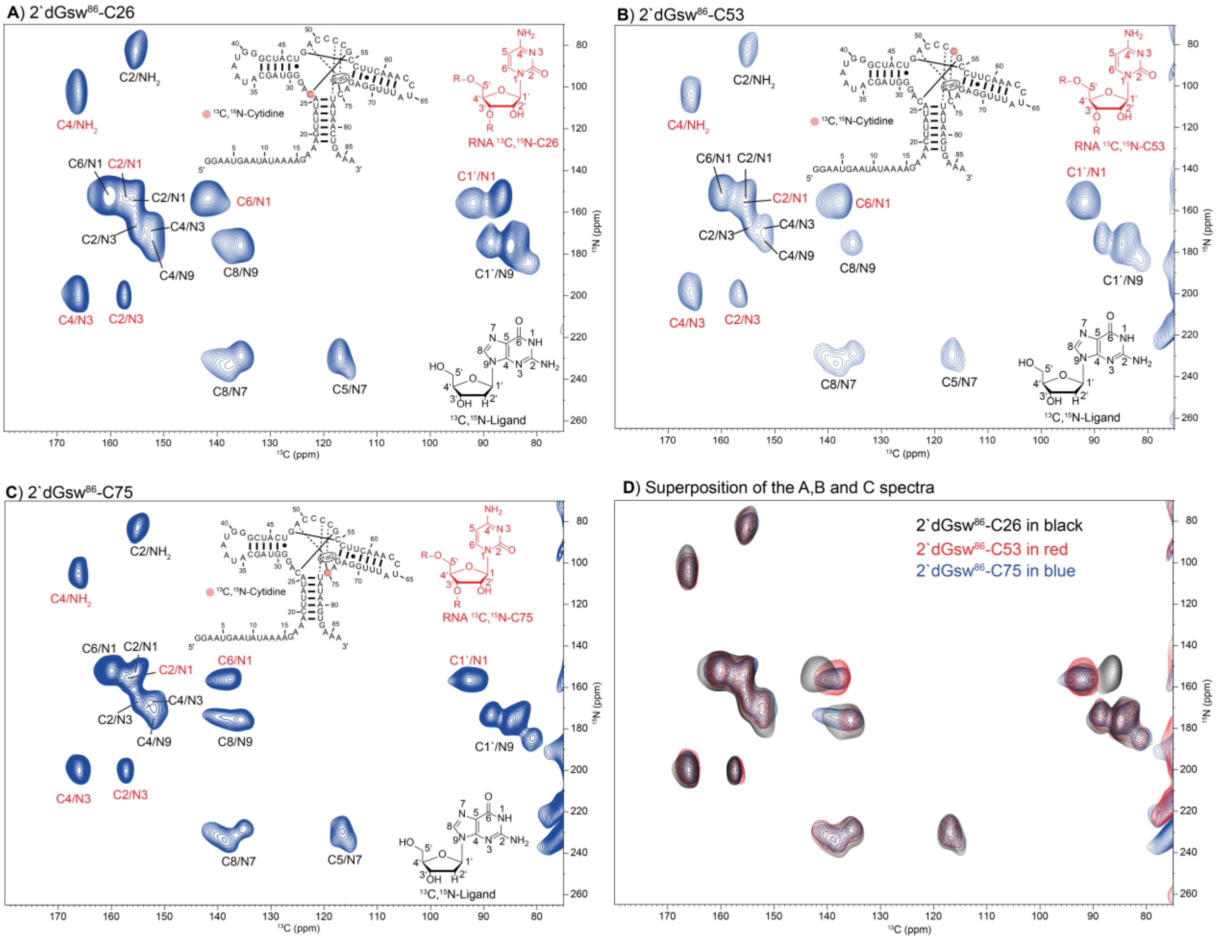

Figure S27 ^{13}C,^{15}N-TEDOR spectra of the 2'dGsw^{86}-C26/C53/C75 RNAs and superposition of these spectra.

5. Solution NMR binding model

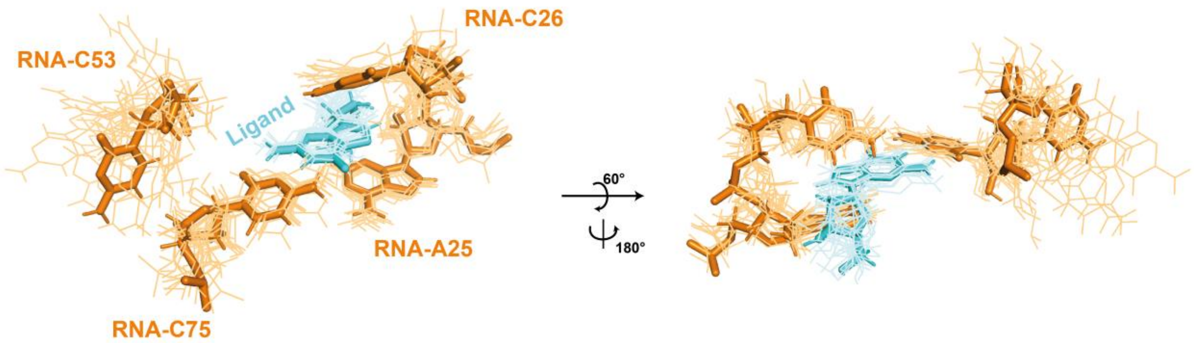

Figure S28 Solution NMR binding pocket model of the RNA 2'dGsw[70] RNA ligand complex.[9] The RNA nucleotides A25, C26, C53 and C75 are shown in orange. Furthermore, additional possible structures are shown as thin lines. The 2'dG ligand is shown in cyan.

6. References

(1) Kao, C.; Zheng, M.; Rüdisser, S. A Simple and Efficient Method to Reduce Nontemplated Nucleotide Addition at the 3' Terminus of RNAs Transcribed by T7 RNA Polymerase. *RNA* **1999**, *5* (9), 1268–1272. https://doi.org/10.1017/S1355838299991033.

(2) *Extinction coefficient online tool*. https://www.fechem.uzh.ch/MT/links/ext.html (accessed 2024-11-19).

(3) *Cytidine-5'-Triphosphate*. https://www.trilinkbiotech.com/cytidine-5-triphosphate.html (accessed 2024-11-22).

(4) Sudakov, A.; Knezic, B.; Hengesbach, M.; Fürtig, B.; Stirnal, E.; Schwalbe, H. Site-Specific Labeling of RNAs with Modified and 19F-Labeled Nucleotides by Chemo-Enzymatic Synthesis. *Chemistry - A European Journal* **2023**, *29* (25). https://doi.org/10.1002/chem.202203368.

(5) Schnieders, R.; Knezic, B.; Zetzsche, H.; Sudakov, A.; Matzel, T.; Richter, C.; Hengesbach, M.; Schwalbe, H.; Fürtig, B. NMR Spectroscopy of Large Functional RNAs: From Sample Preparation to Low-Gamma Detection. *Curr Protoc Nucleic Acid Chem* **2020**, *82* (1). https://doi.org/10.1002/cpnc.116.

(6) Guillerez, J.; Lopez, P. J.; Proux, F.; Launay, H.; Dreyfus, M. A Mutation in T7 RNA Polymerase That Facilitates Promoter Clearance. *Proc. Natl. Acad. Sci.* **2005**, *102* (17), 5958–5963. https://doi.org/10.1073/pnas.0407141102.

(7) Jaroniec, C. P.; Filip, C.; Griffin, R. G. 3D TEDOR NMR Experiments for the Simultaneous Measurement of Multiple Carbon-Nitrogen Distances in Uniformly 13C,15N-Labeled Solids. *J Am Chem Soc* **2002**, *124* (36), 10728–10742. https://doi.org/10.1021/ja026385y.

(8) Güntert, P.; Buchner, L. Combined Automated NOE Assignment and Structure Calculation with CYANA. *J Biomol NMR* **2015**, *62* (4), 453–471. https://doi.org/10.1007/s10858-015-9924-9.

(9) Wacker, A. B. Struktur, Dynamik und Funktion des 2'-Desoxyguanosin-Riboschalters. PhD-Thesis, Goethe University Frankfurt am Main, Frankfurt am Mai, 2012.
